# Longitudinal immune profiling after radiation-attenuated sporozoite vaccination reveals coordinated immune processes correlated with malaria protection

**DOI:** 10.3389/fimmu.2022.1042741

**Published:** 2022-12-15

**Authors:** Fergal J. Duffy, Nina Hertoghs, Ying Du, Maxwell L. Neal, Damian Oyong, Suzanne McDermott, Nana Minkah, Jason Carnes, Katharine V. Schwedhelm, M. Juliana McElrath, Stephen C. De Rosa, Evan Newell, John D. Aitchison, Ken Stuart

**Affiliations:** ^1^ Center for Global Infectious Disease Research, Seattle Children’s Hospital, Seattle, WA, United States; ^2^ Vaccine and Infectious Disease Division, Fred Hutchinson Cancer Center, Seattle, WA, United States

**Keywords:** malaria, vaccine, systems immunology, radiation-attenuated sporozoites, whole blood transcriptional profiling, immunophenotyping, high parameter flow cytometry, serum antibody profiling

## Abstract

**Background:**

Identifying immune processes required for liver-stage sterilizing immunity to malaria remains an open problem. The IMRAS trial comprised 5x immunizations with radiation-attenuated sporozoites resulting in 55% protection from subsequent challenge.

**Methods:**

To identify correlates of vaccination and protection, we performed detailed systems immunology longitudinal profiling of the entire trial time course including whole blood transcriptomics, detailed PBMC cell phenotyping and serum antigen array profiling of 11 IMRAS radiation-attenuated sporozoite (RAS) vaccinees at up to 21 timepoints each.

**Results:**

RAS vaccination induced serum antibody responses to CSP, TRAP, and AMA1 in all vaccinees. We observed large numbers of differentially expressed genes associated with vaccination response and protection, with distinctly differing transcriptome responses elicited after each immunization. These included inflammatory and proliferative responses, as well as increased abundance of monocyte and DC subsets after each immunization. Increases in Vδ2 γδ; T cells and MAIT cells were observed in response to immunization over the course of study, and CD1c+ CD40+ DC abundance was significantly associated with protection. Interferon responses strongly differed between protected and non-protected individuals with high interferon responses after the 1^st^ immunization, but not the 2^nd^-5^th^. Blood transcriptional interferon responses were correlated with abundances of different circulating classical and non-classical monocyte populations.

**Conclusions:**

This study has revealed multiple coordinated immunological processes induced by vaccination and associated with protection. Our work represents the most detailed immunological profiling of a RAS vaccine trial performed to date and will guide the design and interpretation of future malaria vaccine trials.

## Introduction

Currently, there exists a single approved malaria vaccine: the subunit-based RTS,S/AS01 (aka Mosquirix) (1). The RTS,S antigen consists of portions of the circumsporozoite protein (CSP) fused to the viral surface antigen of hepatitis B. However, the efficacy of this vaccine is low: reported as 35.9% (95% CI, 8.1% to 55.3%) in the first year after vaccination for young children (2), and further reduced at later times. Given that the burden of malaria deaths falls most severely on young children in endemic areas, an improved vaccine is urgently needed. Recently, the R21/MM vaccine candidate, also a CSP protein-based vaccine, has shown promise in this area exhibiting 74-77% efficacy (3), however, the study follow-up period was short and the number of individuals enrolled was relatively small.

A key advantage of CSP as a vaccine antigen is that it is highly expressed on the *Plasmodium falciparum* sporozoite, the form of the parasite injected into the skin by mosquito bite that goes on to infect the liver. The sporozoite represents a population bottleneck, as only low numbers of sporozoites successfully infect the liver and go on to induce a symptomatic blood stage infection. Hence, a successful liver-stage specific vaccine would prevent symptomatic disease and transmission and allow the immune system to face a relatively small number of parasites. Whole-sporozoite vaccines represent an alternative approach to CSP-subunit vaccines. Whole-sporozoite vaccines require the administration of entire sporozoites, which are difficult to mass produce compared to a more traditional protein subunit vaccine platform. However, whole sporozoite vaccination presents an enhanced antigenic repertoire while still targeting the low-parasite burden liver stage. It has long been observed that radiation-attenuated sporozoites are capable of inducing sterilizing immunity against live challenge in both animal and human malaria infection (4–7). Along with radiation attenuation, sporozoite administration under anti-parasite chemoprophylaxis (8, 9) and genetic attenuation (10, 11) have also been shown to be effective at preventing infection. Notably, sporozoite administration under chemoprophylaxis has been shown to be capable of eliciting strain-transcendent protection against subsequent challenge (12).

Previous work has described the IMRAS study (13–15), which comprised radiation-attenuated sporozoite vaccination of malaria-naïve individuals who were predominantly male (10 male, 1 female). Studying malaria-naïve rather than malaria experienced individuals allows the clearest picture of the early events involved in protective immunity. It has long been observed that, unlike RAS vaccination, a blood-stage malaria infection will not result in sterilizing immunity to reinfection, but rather a slow development of malaria tolerance after repeated infections (16–19). Blood stage infection is associated with impaired development of immunity to pre-erythrocytic malaria (20, 21).

The IMRAS study was designed with the goal of delivering an intermediate dose of sporozoites that would induce protection in roughly half of the vaccinees, resulting in 55% protection in our study cohort. We have previously published our systems biology analysis of responses to the initial priming immunization in this cohort (13). The most striking protection-associated difference we observed was a sharp increase in type I interferon signaling 1 day after immunization in non-protected individuals. Here, we extend our analysis to a comprehensive set of timepoints after all 5 immunizations and incorporate serum antibody profiling in an attempt to disentangle the complex and highly variable blood transcriptional and cell type abundance dynamics that are associated with RAS-induced protection. We saw that trancriptome responses to RAS differed strongly after each immunization. Populations of multiple cell types including Vδ2 γδT cells and MAIT cells expanded over the course of immunizations. Furthermore, the association of increased interferon with non-protection was specific to immunization 1, with immunizations 2-5 showing a positive correlation with interferon associated gene transcription and protection. Interferon’s association with protection was correlated with different classical and non-classical monocyte abundances after each vaccination.

## Methods and materials

### IMRAS study design

The IMRAS trial (ClinicalTrials.gov NCT01994525) design has been described previously (13–15). In this work, we analyzed IMRAS Cohort 1, which consisted of 11 individuals who received 5 RAS immunizations *via* approximately 200 mosquito bites each. Protective efficacy was determined by CHMI with bites from 5 NF54 *P. falciparum* infected mosquitos. Protected individuals were defined as those that remained free of detectable blood stage malaria infection *via* PCR and thick-blood smear post-CHMI. These vaccinees were accompanied by 3 mock immunized individuals (mosquito bites only) who served as infectivity controls, all of whom were not protected after CHMI.

### Serum antibody profiling *via P. falciparum* antigen protein array

Serum samples were taken from the 11 IMRAS RAS vaccinees and 3 mock-immunized study participants at day 0 (pre-vaccination) and day 140 (3 weeks after the fifth immunization and immediately pre-CHMI). Serum was profiled on *P. falciparum* whole-proteome microarrays, (PfWPM: Antigen Discovery Inc, Irvine CA), consisting of 7,455 *P. falciparum* full or partial proteins, representing 4,805 unique genes (91% of the *P. falciparum* proteome), to quantify levels of *P. falciparum* antigen-specific serum antibodies. *P. falciparum* array antigens were produced using a cell-free ORF-expression clone library followed by *in vitro* transcription/translation (IVTT). After profiling, per-spot microarray signal intensity (MFI) was normalized by taking median foreground intensity minus median background fluorescence intensity, based on IVTT control spots, and log2 transformed.

To identify vaccine-induced serum antibody responses, the normalized MFI response was calculated as the increase in normalized MFI per-participant from day 0 to day 140 for RAS-immunized individuals. Significantly induced antibody responses were identified as those increased at least 2-fold from day 0 to day 140 with an FDR-adjusted single tailed t-test significance threshold of 0.05.

### Whole blood RNAseq

Whole blood RNAseq was performed as previously described (13) on 11 IMRAS Cohort 1 RAS vaccinees. Whole blood was collected directly into PAXgene blood RNA tubes (PreAnalytiX, Hombrechtikon, Switzerland) and stored at −20°C. RNA extraction and globin transcript depletion (GlobinClear, ThermoFisher Scientific, MA, USA) were performed prior to cDNA library preparation using the Illumina TruSeq Stranded mRNA sample preparation kit (Illumina, CA, USA). Globin transcript depletion, cDNA library preparation and RNA sequencing were performed by the Beijing Genomics Institute (Shenzhen, China). A total of sixty-six RNAseq samples were sequenced, with a target depth of 30 million reads per sample. Samples from four timepoints (day 0, 35, 119, 140) were sequenced on Illumina (San Diego, CA) Hiseq2000 sequencers using 75 base-pair (bp) paired-end reads. The remaining samples were sequenced on BGI500 sequencers using 100 bp paired-end reads.

### RNAseq differential expression and gene-set enrichment analysis

Raw RNAseq fastq files were filtered for quality control by adjusting base calls with phred scores < 5 to ‘N’, and read pairs for which either end had fewer than 30 unambiguous base calls were removed. Read pairs were aligned to the human genome (hg19) using STAR (v2.3.1d) (22). Gene count tables were generated using htseq (v. 0.6.0) (23) with the intersection-strict setting on and Ensembl gene annotations (GRCh37.74) used to link genomic locations to gene identifiers. Raw aligned RNAseq counts were normalized to account for different sequencing platforms (Hiseq2000 vs BGI500) using ComBat-seq (24), taking advantage of 4 samples resequenced on both sequencers to ensure an unbiased data transformation. Principal component analysis was performed on log-transformed counts-per-million gene expression data.

The R limma and voom packages (25, 26) were used to identify differentially expressed genes augmented by the dream package (27) to allow the use of a mixed modeling approach. Timepoint and protection were set as fixed effects, including interaction terms, with a random intercept for each participant. For each gene, models were fit using the formula:


geneExpression ∼ protection*timepoint+(1|participant)

To identify genes significantly changed in expression relative to the day of most recent immunization, i.e. vaccine-induced genes, contrasts were constructed of the form ‘timepoint – most recent immunization timepoint’, e.g. ‘D003 – D000’ represents changes at day 3 relative to day 0 (day of first immunization). An FDR cutoff of 0.05, calculated across all contrasts, was used to identify significant genes at all timepoints. Protection-associated genes were identified by F-test, as implemented in the limma topTable function, to identify genes that where any combination of protection:timepoint interaction coefficients were significant.

Gene-set enrichment analysis (GSEA) was performed using the R fgsea package (28), using the BloodGen3 coherently expressed blood transcriptional modules (BTMs) (29). Gene rankings used as input for GSEA were based on estimated log2 fold changes obtained from the limma-dream model. The limma topTable function was used to extract fold changes for each ‘timepoint – most recent immunization induced timepoint’ contrast, which were input to fgsea to calculate vaccine-induced BTM enrichment. Similarly each protection:timepoint interaction coefficient was used to input fold changes into fgsea to identify enriched protection-associated BTMs.

BTM expression was calculated as the 25% trimmed mean of log-transformed counts-per-million gene expression of each BloodGen3 BTM gene, per sample. BTMs with reliable expression, defined as median per-sample CPM > 0, in less than half of their genes were excluded. Non-coherent BTMs, defined as BTMs where less than half of their genes were strongly correlated (Pearson’s r > 0.5) with the trimmed mean of BTM expression were also excluded.

### PBMC flow cytometry profiling

Flow cytometry profiling was carried out using previously published antibody panels and gating strategies (13, 30, 31). PBMCs were collected from IMRAS participants and frozen for later use. After thawing in RPMI supplemented with 10% FBS and benzonase nuclease (Millipore EMD 0.05 U/ml), the samples were incubated with LIVE/DEAD Fixable Blue Dead Cell Stain Kit and the Human BD Fc Block for 30 min at room temperature before being simultaneously stained with four phenotyping panels as detailed in our previous publication (13) (T cell panel), OMIP-044 (30) (DC panel), OMIP-064 (31) (NK panel) and **Datafile S2** (including sample gated B and T cell populations), with markers, antibody clones and fluorophores also listed in **Table S1**. The cells were then profiled using a BD FACSymphony flow cytometer. The data were analyzed, and cellular populations gated and quantified as previously using FlowJo Software (version 9.6.6). The abundance of each manually gated cell subset was calculated as a percentage, using the counts of each defined cell subset divided by the total single live cells from that sample.

### Statistical analysis of flow cytometry cell counts

Mixed models were used to identify vaccine-induced (i.e. significantly changed in abundance over time) and protection-associated cell type subsets using the R lme4 package (32). A nested approach was taken where a ‘full’ model including protection and timepoint as fixed effects, as well as interaction terms, was compared to protection-only and timepoint-only fixed effect models. In all cases, per-participant intercepts were fit as random effects.


FULL: cellTypeAbundance ∼ protection*timepoint+(1|participant)

TIMEONLY:cellTypeAbundance ∼ timepoint+(1|participant)

PROTONLY:cellTypeAbundance ∼ protection+(1|participant)

ANOVA was used to compare the FULL model to the TIMEONLY model (to determine the effect of protection on abundance) and the PROTONLY model (to determine the effect of sampling timepoint on abundance) in a nested manner, and thus calculate associated Chi-squared statistics and accompanying p-values for the reduction in model deviance attributable to adding terms for protection or timepoint. An FDR threshold of 0.05, calculated across all contrasts, was then applied to determine significance.

## Results

### The IMRAS study

The IMRAS study design has previously been described (13–15). Briefly, study participants were immunized five times with radiation attenuated *P. falciparum* NF54 sporozoites (RAS) delivered by mosquito bite, with roughly 200 bites per-immunization. Immunizations were spaced 4-5 weeks apart with protection determined by controlled human malaria infection (CHMI) three weeks after the final immunization. In this study, we analyzed serum antibody levels, whole blood transcriptomics and PBMC cell phenotypic profiles from the 11 RAS-immunized (6 protected, 5 non-protected) participants in IMRAS Cohort 1. The cohort also included three mock-immunized individuals who received sporozoite-free mosquito bites followed by CHMI on the same schedule as RAS vaccinees. Serum, PBMCs and whole-blood PAXgene samples were obtained for up to 5 timepoints per-immunization per-participant for analysis (**Table 1**).

**Table 1:** IMRAS sampling schedule. X indicates where specific sample types were taken.

	Imm 1					Imm 2				Imm 3				Imm 4			Imm 5				CHMI
**Day**	**0**	**1**	**3**	**7**	**14**	**28**	**31**	**35**	**42**	56	**59**	**63**	**70**	**84**	**87**	**91**	**119**	**120**	**122**	**126**	**140**
**Whole blood**	X	X	X	X	X	X	X	X	X	X	X	X	X	X	X	X	X	X	X	X	X
**PBMC**	X		X	X	X					X	X	X	X				X		X	X	
**Serum**	X																				X

Bold indicates the sampling day, e.g.1/3/7 days into the study. Non-bold 'X' indicates whether a particular tissue sample was taken that day.

### RAS immunization induced detectable serum antibody responses

**Figure 1:**
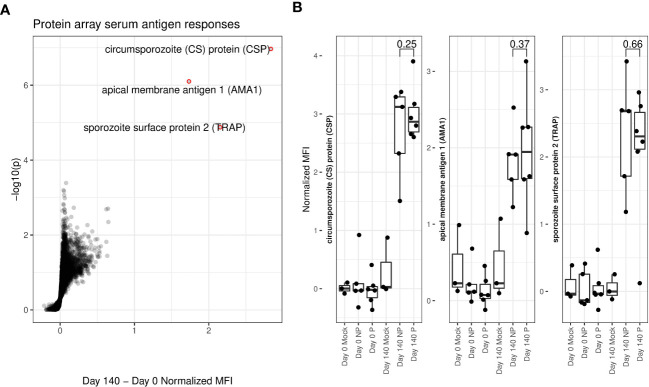
Protein array determined serum antigen responses. **(A)** Volcano plot. X-axis shows increase in protein array normalized mean fluorescent intensity (MFI) from pre-immunization (day 0) to immediately pre-challenge (day 140) timepoints for RAS-immunized participants. Y-axis shows matching log-transformed false-discovery rate (FDR) adjusted single-tailed t-test p-values. Significant (FDR < 0.05) and highly increased (MFI increase > 1) antibody responses are highlighted with red circles and labelled with the corresponding protein. **(B)** Box and dot-plots of pre-vaccine and pre-challenge MFIs for each study participant for labelled antibody responses from **(A)** shown separately for protected (P) and non-protected (NP) RAS vaccine recipients. Mock immunized individuals are included for reference. T-test p-values comparing pre-challenge antibody binding MFIs between P and individuals are indicated.

Serum samples were taken from all 11 vaccinees and 3 mock-immunized individuals immediately prior to immunization (day 0) and challenge (day 140). Each individual serum was screened on *P. falciparum* antigen protein arrays [Antigen Discovery Inc.] to detect the presence of *P. falciparum*-specific antibodies. Strongly significant antibody responses to three *P. falciparum* proteins: circumsporozoite protein (CSP), Apical membrane antigen 1 (AMA1) and thrombospondin related adhesion protein (TRAP) were seen in RAS immunized individuals (**Figure 1A**). All three of these proteins are known to be expressed on the surface of on the apical membrane of *P.falciparum* sporozoites, and are involved in hepatocyte invasion and binding (33–35). Antibody responses did not significantly differ between protected and non-protected individuals (**Figure 1B**).

### Differential expression analysis identified vaccine-induced and protection-associated genes

To identify vaccine-induced responses that correlate with protection, we carried out longitudinal whole blood transcriptomic profiling of IMRAS RAS vaccinees over the entire course of repeated immunizations. A mixed modeling approach was applied (see Methods) to identify genes that were responsive to vaccination and/or associated with protection that also accounted for gene expression levels primarily attributable to per-participant effects. Principal component analysis of transcriptome profiles showed that whole blood expression profiles clustered closely together by participant, rather than by longitudinal timepoint (**Figure S1**), underscoring the need to incorporate transcriptional per-participant effects as a factor in our differential expression analysis.

**Figure 2:**
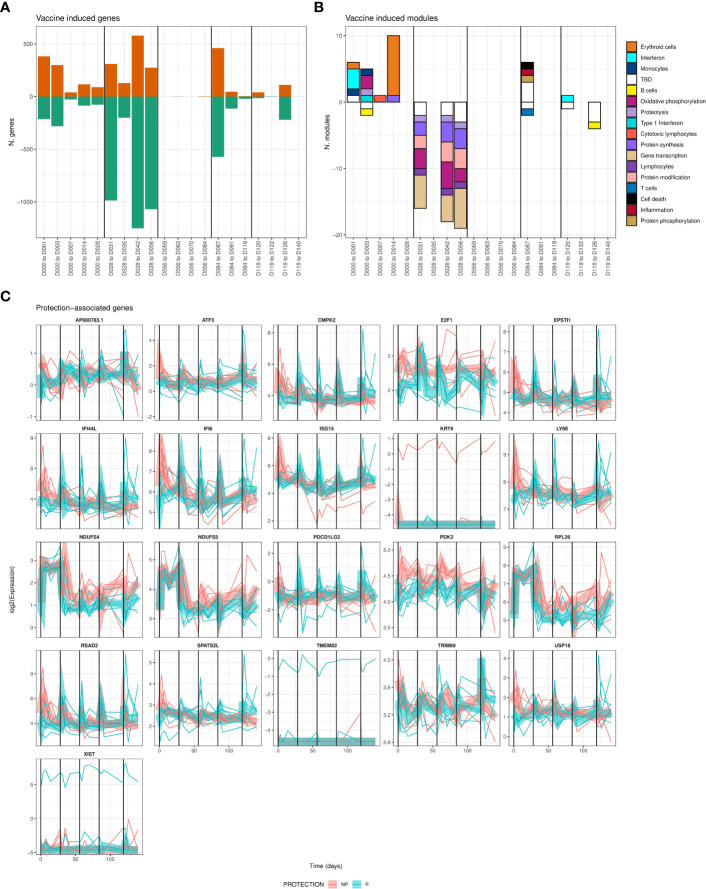
Differentially-expressed genes associated with vaccine-induced and/or protection-associated responses to immunization. **(A)** Bar plot showing counts of genes significantly increased (positive) or decreased (negative bar) relative to the day of the most recent vaccination. **(B)** Bar plot showing counts of BloodGen3 modules enriched in genes shown in A., per-timepoint and direction (positive bars represent modules enriched in genes with increased expression relative to the day of the most recent immunization, negative bars represent enrichment for genes with decreased expression). Module functional annotation is shown by the color key. **(C)** Line plots showing per-individual normalized expression of each significantly (FDR adjusted F-test < 0.05, comparing models including and excluding protection status) protection-associated gene over time colored by protection status. Group-averaged expression is shown as a ribbon. For all panels, vertical black lines indicate times of immunizations.

Vaccine-induced differentially expressed genes (DEGs) were identified as those significantly changed in expression in blood relative to the most recent previous immunization. Vaccine-induced DEGs were identified (FDR < 0.05) at the majority of timepoints across the entire course of immunizations (**Figure 2A**; **Datafile S1**), averaging 471 DEGs at each timepoint for a total of 4112 unique DEGs across all times. Most strikingly, we observed large numbers of genes decreased in expression after the second immunization with over 1,000 DEGs identified at 3 of 4 associated time intervals. In contrast, there were relatively few vaccine induced DEGs, just 4 in total, seen after the third immunization. We quantified the overlap of these DEGs with the recently published BloodGen3 (29) coherent blood transcriptional modules (BTM) to interpret biological processes induced post-immunization. BTMs significantly enriched in vaccine-induced genes that were uniformly increased or decreased per-timepoint are shown in **Figure 2B** (FDR-adjusted hypergeometric p-value < 0.05). Genes increased in expression in the two weeks after the first immunization were enriched for interferon, oxidative phosphorylation and erythroid cell BTMs. Genes reduced in expression after the second immunization were enriched for protein synthesis, protein modification, gene transcription and oxidative phosphorylation BTMs at multiple timepoints. Fewer significant BTMs were identified in DEGs after the 3^rd^ – 5^th^ immunizations, although BTMs representing cell death, and inflammation were increased 3 days after the 3^rd^ immunization. Numbers and expression patterns of DEGs and accompanying enriched BTMs were highly distinct after each immunization.

We also found 21 genes significantly associated with protection when considering all timepoints together (**Figure 2C**). The 21 protection-associated genes significantly overlapped (FDR-adjusted hypergeometric p = 3 x 10^-8^) with a single BTM: six protection-associated genes (EPSTI1, IFI44L, IFI6, ISG15, LY6E, RSAD2) are members of BTM M8.3 representing Type I Interferon responsive genes. These genes were also increased in expression immediately post-immunization, but whereas non-protected individuals had high and sustained increased expression of these genes after the first immunization, protected individuals had stronger increases after the remaining 4 immunizations (**Figure 2C**). This suggests that while interferon responses are broadly associated with protection during RAS immunization, the effect of increased interferon gene expression on protection may change over the course of vaccination. 13 of these 21 total protection-associated genes and 5 of 6 type I interferon protection-associated genes were also significantly vaccine-induced for at least one timepoint.

### Gene-set enrichment analysis revealed complex timing of vaccine responses

**Figure 3:**
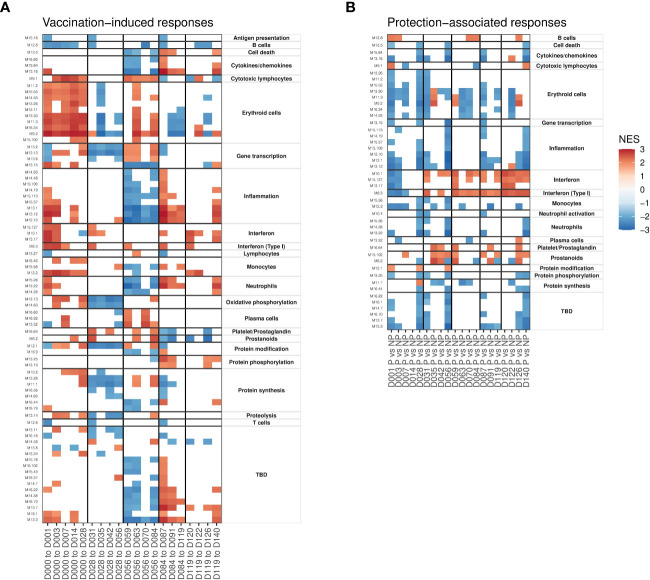
Gene set enrichment analyses showing patterns of vaccine-induced and protection-associated responses profiled using blood transcription modules (BTMs). Heatmaps show significant (FDR < 0.05) normalized enrichment scores (NES). **(A)** BTMs significantly enriched for genes changing in expression relative to day of the most recent immunization. **(B)** BTMs significantly enriched for genes with expression differences between protected and non-protected individuals at each timepoint. Vertical black lines indicate times of immunizations in both panels.

Modules identified as having enriched overlap with DEGs were limited by the binary significance cutoff used to identify DEGs. Therefore, we applied gene-set enrichment analysis (GSEA) to identify BTMs that were significantly enriched in the full rank-ordered gene list. Genes were ranked based on the above determined linear model coefficients and normalized enrichment scores (NES) calculated to classify BTMs as being increased or decreased relative to the day of the most recent immunization (**Figure 3A**) or associated with protection at a specific timepoint (**Figure 3B**). Overall, vaccine-induced responses identified as enriched in DEGs (**Figure 2B**) were also captured in the GSEA analysis; for example, increased interferon and erythroid cell BTMs after the first immunization, and decreased gene transcription and protein synthesis BTMs after the second immunization. However, the vaccine-induced GSEA captured many events after the third, fourth and fifth immunizations not seen in the DEG-based analysis. This includes increases in erythroid cell and gene transcription and decreased inflammation after the third immunization as well as increased inflammation, interferon and protein phosphorylation after the fourth and fifth immunizations. Many significantly enriched BTMs fall into the ‘TBD’ category, where the overall functional annotation was not determined. These show enrichment patterns similar to inflammation and cytokine/chemokine BTMs, suggesting co-ordination with defined immune responses. Concordant with the individual DEG analysis, we saw highly distinct response patterns induced after each immunization.

Focusing on protection-associated BTMs, we saw more consistent patterns after each immunization. The majority of protection-associated processes comprised BTMs annotated as associated with specific immune cell types (adaptive and innate immune cells), cytokines, erythroid cell, and inflammation and proliferation (protein synthesis/phosphorylation/modification) that were consistently reduced in protected individuals relative to non-protected individuals 1-3 days after each immunization (**Figure 3B**). As with the vaccine-responsive BTMs above, ‘TBD’ modules mimicked the protection-associated enrichment patterns of inflammation associated BTMs. Interferon and prostanoid BTMs were increased in protected participants immediately after immunizations 2-5, but not after the first immunization. These interferon responses are consistent with what was observed in the DEG analysis above.

### Hierarchical clustering showed distinct patterns of protection-associated responses

**Figure 4:**
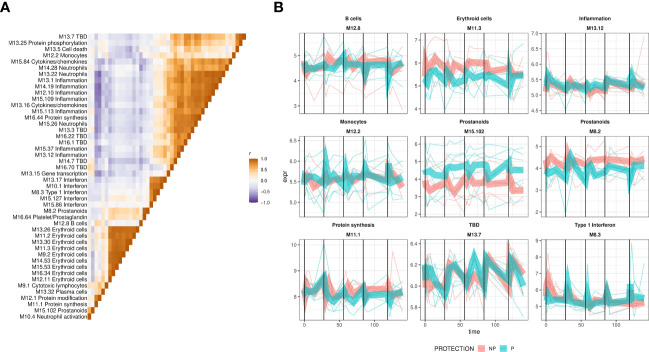
Expression dynamics of protection-associated BTMs. Expression of protection-associated BTMs identified by GSEA was calculated as the average of BTM gene expression and used to create a BTM correlation matrix. **(A)** The BTM correlation matrix. Expresison of protection associated BTMs, identified by GSEA, was calculated as the average of BTM gene normalized expression, and pairwise Pearson correlation coefficients calculated and used as a basis for hierarchical clustering of the matrix. Colors show BTM-BTM correlation coefficients (Pearson’s r), and BTMs ordered identically on the x- and y-axes by hierarchical clustering of the correlation matrix. **(B)** Averaged expression of representative BTMs from subclusters in **(A)** plotted with lines representing individuals BTM expression, colored by protection status. Protection group average expression shown as thick ribbons.

In order to decipher the different patterns of protection-associated responses, we calculated average BTM expression and hierarchically clustered the modules based on their correlation matrix. BTMs that shared a functional annotation tended to cluster closely (**Figure 4A**) with Interferon BTMs and erythroid cell BTMs forming highly distinct clusters. We also observed a distinct cluster that represented inflammation/neutrophil/cytokine BTMs. This indicated that BTMs representing similar functional annotations shared coherent expression patterns. Representative BTMs were then selected from these sub-clusters and their averaged expression was plotted (**Figure 4B**). Alongside the previously noted type I Interferon BTM, we observed a variety of protection-associated expression patterns, including both transient responses to vaccination and sustained protection-associated differences over the study. Notably, M12.2 monocyte BTM expression showed strong increases in protected individuals shortly after immunizations 2-5. In contrast, the M11.3 erythroid cells and M8.2 prostanoid BTMs showed sustained increased expression in non-protected individuals throughout the study. Thus, GSEA revealed numerous functional gene sets and expression dynamics that were correlated with protection.

### Monocyte, dendritic cell and invariant T cell populations were responsive to RAS vaccination

To better understand the cellular RAS vaccination response profile, we performed high-parameter flow cytometry profiling of PBMCs taken from the IMRAS vaccinees at timepoints overlapping with whole blood gene expression (**Table 1**). Four protein marker panels were used, consisting of 21-28 markers each, specific for B cells, T cells, antigen-presenting cells, and NK/ILC/γδT T cells (**Table S1**). An extensive gating strategy was applied (see Methods) to determine counts for hundreds of different cell types and corresponding activation states (**Datafile S2**). As with whole-blood transcriptomics, cell abundance profile variability was most strongly predicted by person-to-person variability (**Figure S2**) rather than by vaccine-induced changes, which necessitated a mixed-modeling approach to account for this variability while identifying vaccine-induced cell subsets.

**Figure 5:**
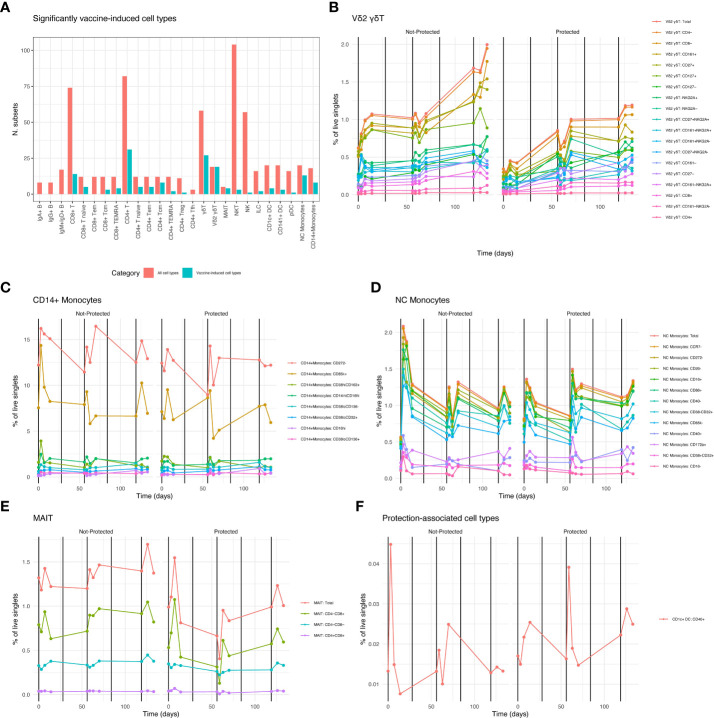
RAS Vaccine-induced and protection associated cell types. **(A)** Counts of significantly (FDR < 0.05) vaccine-induced cell types grouped by lineage and/or function. Red columns indicate total numbers of gated subsets in the corresponding group, adjacent teal columns show proportion of gated subsets that were vaccine-induced. B-E Averaged counts of vaccine-induced cell types showing vaccine induced cell types from specific lineage/function groupings in A., over time. Vertical black lines indicate vaccination times. Legend shows color for each gated cell type subset, ordered from most abundant (top) to least abundant (bottom). **(B)** Vδ2 γδT cells, **(C)** Classical CD14+ monocytes, **(D)** Non-classical (NC) monocytes, **(E)** MAIT cells. **(F)** Averaged counts of the protection-associated (FDR < 0.05) cell type: CD1c+ CD40+ DCs.

Mixed-modeling revealed a total of 174 vaccine-induced cell types (**Figure 5A**; **Datafile S3**) including 19 Vδ2 γδ T (**Figure 5B**) cell subsets, 8 classical CD14+ monocyte subsets (**Figure 5C**), 13 non-classical (NC) monocyte subsets (CD11c+ HLA-DR+ CD16+) (**Figure 5D**) 4 MAIT cell subsets, (**Figure 5E**), along with 48 CD4+ and CD8+ T cell subsets. We observed few or no changes in B cell, NK cell or NKT cell subsets. Several cell type groupings showed coherent changes in abundance over the course of vaccination: NC monocyte subsets were rapidly and transiently increased after immunizations 1 and 3 and 5 in both protected and non-protected individuals. Larger increases were apparent in non-protected individuals after the first immunization with smaller increases after the third immunization, while the opposite pattern was observed in protected individuals (**Figure 5B**). In contrast, Vδ2 γδ T cell subsets continuously increased over the course of vaccination and this increase was more apparent in non-protected individuals (**Figure 5C**). Similar to NC monocytes, classical CD14+ monocytes subsets showed transient increases after each immunization (**Figure 5D**). CD272- (a.k.a. BTLA: B-and T-lymphocyte attenuator) and CD85k+ (a.k.a. LILRB4, leukocyte immunoglobulin-like receptor B4) CD14+ monocytes represented the most abundant induced monocyte populations. For both CD272- and CD85k+ monocytes, increased abundance was apparent at the earliest post immunization 1 timepoint (day 3) in non-protected individuals, but not until day 7 post-immunization for protected individuals.

A distinct pattern was observed for MAIT cell abundance. Total MAIT cell abundance was generally increased after each immunization for both protected and non-protected individuals. However, increases in total and CD8+ MAIT cells were stronger and more rapid in protected individuals after the first immunization. (**Figure 5E**). Vaccine-induced T cell subsets, both CD4+ and CD8+, were not confined to a single memory or effector phenotype. Since malaria sporozoite-specific T cells represent a small (or zero) proportion of total T cells it is unclear what is driving changes in circulating T cell proportion. These changes may simply reflect changes in the total number of circulating myeloid cells in response to vaccination.

It is important to note that none of the protection-associated trends listed in the vaccine-induced cell types described above reached statistical significance. We identified a single significantly protection-associated cell type: CD40+ CD1c+ DCs (**Figure 5F**). These showed a similar overall expression pattern as the NC monocytes, but with a more pronounced increase in abundance in non-protected individuals after the first immunization and a more pronounced spike in protected individuals after the third immunization. In summary, we observed that myeloid cell types including classical CD14+ monocytes, non-classical monocytes and DCs tended to show similar vaccine response and protection dynamics as type I interferon-associated gene expression, while other vaccine responsive lymphoid cell types such as MAITs and Vδ2 γδT cells had distinct response patterns over time.

### Combined analysis of cell populations and gene expression revealed cell types correlated with vaccine-induced and protection-associated transcriptional responses

**Figure 6:**
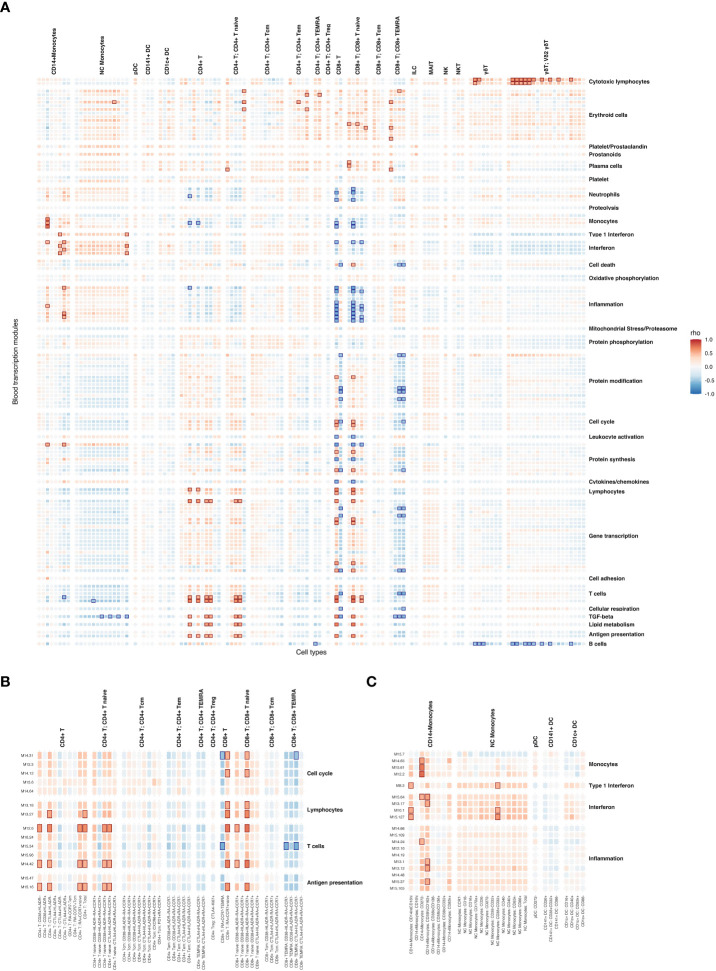
Correlations between vaccine-induced cell type abundances and vaccine-induced BTM expression. **(A)** Heatmap showing Spearman correlations between vaccine-induced cell type abundance and average BTM expression. Only BTMs with at least one significant cell type correlation are shown. Strong positive and negative (|ρ| > 0.5 & FDR < 0.05) correlations are highlighted with red and blue boxes respectively. **(B)** Subset of heatmap **(A)** with T cell subsets labelled. **(C)** Subset of heatmap **(A)** with DC and Monocyte subsets labelled.

To better understand the relationships between cell type abundances and transcriptional responses after RAS immunization, we created a comprehensive correlation map relating RAS induced BTM expression to cell type abundances. Correlations between BTM expression and vaccine-induced cell type abundances were calculated by matching transcriptomic profiles with flow cytometry derived cell type abundance from each participant at shared timepoints (**Table 1**). Multiple significant cell type-BTM correlated pairs were observed (**Figure 6A**), and these correlations often reflected known functions of the cell type in question. For example, BTMs with the ‘inflammation’ functional annotation were correlated with CD14+ monocyte subsets and BTMs with the ‘cytotoxic lymphocyte’ annotation were correlated with γδ T cells. Many BTMs functionally annotated as ‘antigen presentation’, ‘lymphocytes’; ‘T cells’, and ‘cell cycle’ were specifically correlated with naïve CD4+ and CD8+ T cell subset abundance (**Figure 6B**), possibly reflecting initiation of adaptive responses post immunization.

Several monocyte subsets were significantly (FDR < 0.05) and strongly positively correlated (Spearman’s ρ > 0.5) with Interferon BTM expression, specifically the CD16hi, CD272- and CD38hiCD163+ subsets of CD14+ monocytes, and CD38+CD32+ NC monocytes (**Figure 6C**). Vaccine-induced NC monocyte subsets were uniformly positively correlated (all NC monocyte – interferon ρ > 0.2) with all 4 interferon BTMs. While most CD14+ monocyte populations were positively correlated with interferon BTMs, this was not always the case for CD38loCD32+ or CD16hi populations of CD14+ monocytes. Therefore, it appeared that NC monocyte abundance was linked to interferon expression in the blood across the entire study period regardless of NC monocyte activation/maturity status, while this was not the case for CD14+ monocyte populations.

To further explore the temporal linkage between interferon transcriptional responses and DC/monocyte abundance, we investigated how the strength of their correlation varied over time. After immunization 1 (day 0-28) specifically, 2 CD14+ monocyte subsets were significantly positively correlated with interferon BTM expression. In contrast only 1 NC monocyte subset and no CD1c+ DCs (**Table 2**) were correlated with interferon BTMs. This changed after immunization 3, when 9 NC monocyte subsets, including total NC monocyte counts, were significantly correlated with interferon along with 3 CD14+ monocyte subsets and one CD1c+ DC subset (the protection associated CD1c+ CD40+ DC subset). This suggests that non-protection associated interferon responses after immunization 1 are primarily associated with subsets of CD14+ monocytes, while protection-associated interferon responses after immunization 3 were correlated with increased abundance of a wider spectrum of both CD14+ and NC monocyte populations.

**Table 2:** Numbers of DC/monocyte subsets significantly (FDR < 0.05) and positively (rho>0.25) correlated with at least one interferon BTM after each immunization.

	All timepoints	Immunization 1	Immunization 3	Immunization 5
	(Day 0 -140)	(Day 0 - 28)	(Day 56 - 81)	(Day 119-140)
**CD14+Monocytes**	CD14intCD16hi	CD272-	CD14intCD16hi	
	CD272-	CD38hiCD163+	CD272-	
	CD38hiCD163+		CD38hiCD163+	
	CD85k+			
**CD1c+ DC**	CD16+		CD40+	
	CD40+			
**NC Monocytes**	Total	CD38+CD32+	Total	
	CCR7-		CCR7-	
	CD16-		CD16+	
	CD16+		CD26-	
	CD26-		CD272-	
	CD272-		CD38+CD32+	
	CD38-CD32+		CD40+	
	CD38+CD32+		CD85k-	
	CD40-		CD86+	
	CD40+			
	CD85k-			
	CD86+			

## Discussion and conclusions

Radiation-attenuated sporozoite vaccination is known to induce sterilizing protection against malaria, and many studies have investigated its protective efficacy and immune consequences (13, 14, 36–39). Our work stands out as the most comprehensive profiling to date of radiation-attenuated sporozoite immunization and challenge in malaria naïve individuals. Previously (13), we reported that increased abundance of type I interferon-associated genes in blood were associated with a lack of protection in malaria-naïve RAS vaccinees after the first immunization. Here we extend our analysis by showing that interferon responses are subsequently increased more strongly in protected relative to non-protected individuals after subsequent boosting immunization. We also identified several other independent transcriptional pathways associated with RAS-induced malaria protection. Furthermore, high levels of interferon-associated gene transcription in blood after the first immunization were correlated with a small number of specific classical CD14+ monocyte subpopulations, while peripheral interferon responses to subsequent immunizations were correlated with increased abundance of broader range of classical and non-classical monocytes, along with CD1c+ dendritic cells.

Serum antibody profiling revealed robust serum antibody responses to abundant sporozoite surface antigens, i.e. CSP, TRAP and AMA1, that were vaccine-induced but not protection-associated. We did not see significant changes in B cell subset abundances in blood induced by vaccination, or associated with protection, likely reflecting small total numbers of *P. falciparum* antigen specific B cells in circulation. The relationship between malaria protection and neutralizing antibodies is complex: despite a clear role for antibodies in protection against malaria, the association of serum antibody levels with protection is inconsistent (40, 41). It has been shown that malaria protection can be elicited by sufficiently high levels of neutralizing antibodies delivered *via* passive transfer (42), but It is likely that the antibody levels elicited in this IMRAS study cohort were insufficiently high to achieve protection alone, and require the participation of other mechanisms, such as malaria specific CD8+ T cells (43, 44). Also, our study is likely underpowered to discover different potential routes of protection from alternate antigens and to capture the breadth of antigen responses on an individual level. We did not explore Fc mediated antibody function, which has been previously associated with protection (40).

The biggest factor driving variability in transcriptome and circulating cell phenotype populations was individual variability. Each vaccinee occupied a distinct region of the dimensionality-reduced transcriptomic space (**Figures S1**, **S2**), illustrating that per-participant variability has a stronger effect than responses induced by vaccines. It is reasonable to assume that our limited sample size has not extensively explored common human transcriptomic phenotypes, and further insights into RAS immunity would be accessible by profiling larger cohorts. A further set of 10 IMRAS RAS vaccination and challenge samples from Cohort 2 (15) were also available, however Cohort resulted in 9/10 individuals being protected post CHMI. Having only one participant in the non-protection outcome group meant we lacked statistical power to validate our transcriptomic or flow cytometry results in this group. Since our linear mixed modeling approach incorporates a per-individual effect in our models, the scope of statistical inference is limited to these individuals, thus further underlining the need for independent validation of these results. Nevertheless, significantly altered transcriptome responses and cell type abundances associated with vaccination and protection were evident. Vaccine-induced responses were highly distinct after each vaccination. We identified multiple coherently expressed transcriptional responses significantly responsive to immunization. Responses associated with vaccination, but not protection, primarily comprised strong reductions in proliferative processes (protein synthesis, gene transcription, protein phosphorylation) and oxidative phosphorylation BTMs after immunization 2. Metabolic processes in whole blood reflect the sum of each individual cell types’ metabolic program. As immune cells comprise the vast majority of nucleated blood cells, changes in metabolic gene expression in blood reflect changes in immune cell metabolism. Overall, vaccination-associated processes seem to represent broad shifts in immune cell metabolism and abundance after vaccination. Immature neutrophils use oxidative phosphorylation (OXPHOS) for energy production, while mature neutrophils rely on glycolysis (45). Additionally, classically activated M1 macrophages and DCs upregulate glycolysis in preference to OXPHOS (46). Thus, the reduction in OXPHOS and proliferative signaling after immunization 2 may reflect the increased presence of mature innate cell phenotypes. Additionally, OXPHOS is associated with anti-inflammatory M2 macrophages, while pro-inflammatory M1 macrophages rely on glycolysis (47). Therefore, this reduction in OXPHOS after immunization 2 but not immunization 1 may reflect a shift in the balance of M1 vs M2 macrophages in the skin/site of infection after the first immunization. Alternatively, changes in OXPHOS signaling could be driven by T cell activation, as quiescent naïve and memory T cells primarily rely on OXPHOS, however activation *via* TCR stimulation causes a shift to glycolysis (48). The large numbers of vaccine-induced genes observed immediately post-immunization contrasts with a recent whole-sporozoite with chloroquine prophylaxis vaccine study (CPS) with a similar number of participants and protective efficacy (8). This is likely driven by the much smaller dose of sporozoites delivered: in the CPS study subjects received 5-15 bites from infected mosquitos per immunization vs. approximately 200 in IMRAS. However, BTMs implicated in protection largely overlapped between studies, including monocytes, neutrophils, inflammation and interferon.

Professional antigen presenting cells have an essential role in the development of vaccine-induced immunity, thus it is not surprising that monocyte and DC abundances were clearly induced by vaccination. In particular, multiple NC monocyte (30) subsets were increased after vaccination. In the original OMIP publication of the flow panel, these NC monocytes are referred to as double negative (DN) DCs, i.e. CD141- CD1c- CD14- CD11c+ cells which do not fall into the classical cDC1 or cDC2 classes and are not pDCs. However, we observed that almost all of these ‘DN DCs’ are CD16+. Previous work has shown that CD14dim CD16+ CD11c+ CD1c- CD141- cells are better described as non-classical monocytes and not DN DCs (49, 50). Thus, our results have consistently referred to non-classical (NC) vs. classical CD14+ monocytes. We saw increased abundance of classical CD14+ monocytes after the first immunization followed by increases in a broad mix of classical and non-classical monocytes after immunizations 3 and 5.

Along with myeloid cells, RAS vaccination was associated with changes in non-conventional T cell populations. We saw increases in MAIT cell abundances in response to RAS immunization with a tendency towards larger increases in protected individuals. Mpina, et al. (51) have shown a long-lived, dose-dependent increase in MAIT cells following resolution of infection after CHMI with *ex-vivo* experiments that associated MAIT activation with blood stage malaria parasitemia, but not RAS administered by direct venous injection. It is therefore possible that MAIT cell responses detected here reflect immune responses to the mosquito bite, however this would not account for a trend towards larger increases in protected individuals, suggesting further investigation of the role of MAIT cells in malaria protection. Vδ2 γδT cells have been previously shown to be intrinsically activated by malaria (52, 53) and elevated in protected individuals after whole-sporozoite vaccination (54). We observed a consistent increase in Vδ2 γδT cell counts over the entire course of vaccination and non-protected individuals trended towards larger cumulative increases in these cells. This was particularly apparent after the fifth immunization, although this difference was not statistically significant. While this may appear at odds with the described protective role of Vδ2 γδT cells, other work suggests a complex relationship between Vδ2 γδT and malaria immunity: it has been reported that a decrease in Vδ2 γδT cells levels is associated with tolerance (55) and reduced severity of malaria infection (56).

We previously observed a detrimental role of type I interferon in the development of malaria protection after prime immunization and a correlation between it and non-classical monocytes. In the current study, we have further explored the dynamics of interferon response following RAS vaccination and observed that type I interferon was negatively associated with the initial establishment of adaptive memory, but positively correlated with memory expansion upon boosting. We note that our measurements of type I IFN responses were transcriptional, and we did not directly measure protein levels, e.g. secreted IFN induced cytokines, as our serum sampling timepoints (**Table 1**) were either pre-immunization (day 0), or 3 weeks after immunization 5. However, we also saw that these interferon responses were significantly correlated with monocyte frequency, consistent with our previous findings. A potential explanation for this phenomenon is that over-induction of type I interferon inhibits the initial establishment of memory cell populations. Sporozoite antigens are taken up and presented by DCs after caspase-mediated cell death (57), and type I interferon can inhibit caspase activity (58) and hence antigen presentation. The detrimental role of interferon in the development of CD8+ T cell mediated immunity has also been observed in animal models (44). It remains to be seen whether the role of interferon in is specific to RAS immunization or is relevant to the development of human liver-stage malaria immunity more broadly. Radiation-attenuated sporozoites cannot replicate in the liver resulting in incomplete liver stage development without expansion of parasite mass. This is different in key respects to other whole-sporozoite vaccination approaches such as vaccination using late-arresting genetically attenuated sporozoites (11) or administration of live sporozoites with anti-malarial prophylaxis (8, 12), which allow liver stage parasite expansion. Thus, the arrested nature of the RAS liver stage may explain the transient nature of the IFN transcriptional responses that peaked 1-3 days post immunization. In contrast to these results, previous work investigating transcriptional responses to RTS,S in malaria naïve individuals (59) did not observe any association between initial immunization and IFN transcriptional responses, although, consistent with our work, they did observe a positive association between IFN responses 1-5 days after the 2^nd^ immunization and subsequent protection. We hypothesize that these early IFN differences may be due to a stronger innate response to a whole-sporozoite RAS vaccination vs. the subunit RTS,S vaccine. Our previous work looking at malaria-experienced RAS vaccinees (38) also did not observe differences in transcriptional IFN responses to immunization between protected and non-protected individuals, however we did observe an overall reduction in the proportion of protected individuals, and a large proportion of non-protected BSPZV1 participants had elevated expression of IFNα response genes prior to initial immunization, suggesting a role for chronic inflammation in dampening RAS vaccine efficacy.

While abundances of CD14+ and NC monocyte populations were not significantly associated with protection, different monocyte subsets were correlated with interferon transcriptional responses after immunizations 1, 3 and 5, suggesting that the distinct protection-associated roles of interferon after immunization 1 vs immunizations 2-5 may reflect the balance of classical vs. non-classical monocytes responding to vaccination. Specifically, protection-associated interferon transcriptional responses were correlated with increased levels of both CD14+ and NC monocyte subsets, while the non-protective interferon responses after the 1^st^ immunization were correlated with CD14+ subsets only. Interestingly, increased abundance of non-classical CD14dim CD16+ monocytes has been previously linked to reduced serum inflammatory cytokine levels and less severe malaria in children (60), pointing to a key role for these monocyte subsets in resolving the inflammatory response to malaria. Thus, NC monocytes may play an immune regulatory role in response to RAS immunization, and effective vaccine mediated immunity may require a balance between classical and non-classical monocyte responses. The sole protection-associated cell type we identified was CD40+ CD1c+ DCs, which are activated cDC2 cells. cDC2s are the predominant DC population in blood, and can cross present to and potently activate Th1, Th2, Th17 and CD8+ T cells (61). CD40 is a co-stimulatory molecule that plays a central role in T and B cell activation and regulation when expressed on DCs (62). CD40 has been shown to be required for liver-stage protective immunity to malaria infection in a mouse model (63). It has also been observed that blood derived DCs upregulated CD40 upon exposure to P. falciparum infected red blood cells (64). CD40+ CD1c+ DC abundances were also correlated with interferon responses across all timepoints and specifically after immunization 3 in pattern similar to many NC monocyte subsets, suggesting that NC monocytes and DCs may play a coordinated role mediating expansion of adaptive memory responses.

Apart from interferon-associated signaling, BTMs associated with inflammation, cytokines and chemokines, monocytes, neutrophils, and erythroid cells were identified as protection associated by GSEA. In general, protected individuals showed reduced induction of these processes immediately after every immunization, distinct from what we saw with interferon. However, it is difficult to disentangle the specific role each of these individual processes play in protection. Interestingly, we have seen that increased pre-vaccination abundance of BTMs associated with inflammation, monocytes, and neutrophils was associated with protection against malaria challenge (65), raising the possibility that the reduced responses seen in protected individuals post-immunization is due to their higher baseline expression of these modules, giving them an immunological ‘head start’ in responding to immunization more broadly. In contrast to the large numbers of significantly vaccine induced genes identified in this work, our previous pre-vaccination analysis (65) of correlates of protection did not identify specific genes significantly associated with protection, but rather coordinated differences in gene set expression in protected individuals. The observation that both differences in inflammatory states pre-vaccination and inflammatory responses to vaccination contribute to protection suggests that vaccination acts as an immune stimulus that allows subtle underlying pre-dispositions to protection to be revealed. That hypothesis is supported by a recent cross-study analysis of 13 different vaccines showing that a heightened inflammatory state prior to immunization marked by increased expression of monocytes, dendritic cells and pro-inflammatory genes was associated with higher serum antibody responses across vaccines (66). Multiple BTMs associated with erythroid cells were identified as increased and negatively associated with protection 1-3 days post-immunization after the first three immunizations. It is not immediately clear why erythroid cell-associated processes might be important for RAS-mediated protection, but there are several possible explanations. For example, increased erythroid cell BTMs may reflect anemia of inflammation (67) where inflammation induces upregulation of hepcidin in the liver, resulting in iron retention in macrophages and reduction in erythroid cell differentiation and proliferation *via* inhibition of erythropoietin. Also, it has been shown that type I interferon is involved in hematopoiesis (68, 69) and activates dormant hematopoietic stem cells. Increased prostaglandin BTMs were also associated with protection and their role may intersect with erythroid cell signaling, as free heme impairs the anti-inflammatory prostaglandin E_2_ (70) while decreased levels of plasma-circulating prostaglandin E2 were correlated with more severe malaria disease in children (71).

Taken together, our systems immunology profiling results demonstrate that a finely tuned and coordinated set of immune responses are involved in establishing protection after RAS immunization, in particular the interplay between interferon transcriptional responses and classical and non-classical monocyte abundances. These also include responses previously reported, and observed here, to be induced by whole sporozoite vaccines (e.g. expansion of Vδ2 γδT cells) and more general inflammatory, antigen uptake and presentation responses necessary for development of immunity from any vaccine. This work has revealed that the coordination of protection-associated peripheral blood responses is complex, highly variable on an individual level and distinct after each vaccination. Although these results have yet to be validated in a comparable independent vaccine cohort, we hope that the urecedented depth and resolution of our analyses, and the public availability of the accompanying IMRAS profiling data, will inform future design and interpretation of whole-sporozoite vaccine trials.

## Data availability statement

Whole blood transcriptional data is available on NCBI GEO under accessions GSE116619 (Days 0-28) and GSE192756 (Days 31-140). Antibody microarray profiling results and flow cytometry subset counts are available as Datafiles S 4 and S 5 respectively, with raw data available in ImmPort.

## Ethics statement

The study was conducted at the Naval Medical Research Center (NMRC) Clinical Trials Center from 2014 to 2016; controlled human malaria infections (CHMIs) were conducted at the Walter Reed Army Institute of Research (WRAIR) secure insectary. The study protocol was reviewed and approved by the NMRC Institutional Review Board in compliance with all federal regulations governing the protection of human subjects. WRAIR holds a Federal-wide Assurance from the Office of Human Research Protections (OHRP) under the Department of Health and Human Services as does NMRC. NMRC also holds a Department of Defense/Department of the Navy Federal- wide Assurance for human subject protections. All key personnel were certified as having completed mandatory human subjects’ protection curricula and training under the direction of the WRAIR Institutional Review Board or the NMRC Office of Research Administration (ORA) and Human Subjects Protections Branch (HSPB). All potential study subjects provided written, informed consent before screening and enrollment and had to pass an assessment of understanding. This study was conducted according to the Declaration of Helsinki as well as principles of Good Clinical Practices under the United States Food and Drug Administration Investigational New Drug (IND) application BB-15767. This trial was performed under an IND allowance by the Food and Drug Administration (FDA).

## Author contributions

FD designed and performed computation analysis and integration with assistance from YD, MN, DO, and NM. Flow cytometry was performed by NH, KVS, and SD. Wet lab work on RNAseq and serum profiling was performed by JC and SM. Study conceptualization and funding and supervision was by EN, MJM, JA, and KS. Manuscript drafting was done by FD and all authors contributed to editing, revising and approving the final manuscript.

## Supplementary material

Supplementary Figure 1 Principal component analysis plots of normalized whole blood gene expression data. Each panel shows colored points corresponding to all timepoints from a single participant with samples from other study participants shown in grey.

Supplementary Figure 2 Principal component analysis plots of cell-type profiles determined by flow cytometry. Each plot panel shows the first two principal components per-sample from flow panels for B-cells; CD3+ T cells; NK and invariant T-cells; and DCs and other antigen presenting cells respectively. Each sub-panel is labelled and colored to highlight a single study participant, with the remaining samples shown in grey.

Supplementary Table 1 Flow cytometry panel marker panels. Each column lists the protein markers used in each flow cytometry panel in this study.

Supplementary Data Sheet 1 Limma-dream results table of significant (FDR < 0.05) vaccine-induced genes.

Supplementary Data Sheet 2 Gating diagrams for each flow cytometry panel, plus gated flow cytometry plots for the T and B cell panels, with parent gated populations indicated.

Supplementary Data Sheet 3 Mixed-model p-values and high-level cell type grouping for each vaccine-induced cell type.

Supplementary Data Sheet 4 Serum antibody array mean fluorescent intensity (MFI) array profiling data for each antigen and sample, normalized relative to IVTT controls.

Supplementary Data Sheet 5 Gated flow cytometry count table, annotated with cell types for each participant at each timepoint.

## References

[1] Kester KE Cummings JF Ofori-Anyinam O Ockenhouse CF Krzych U Moris P. S/AS01B and RTS,S/AS02A in malaria-naive adults: safety, efficacy, and immunologic associates of protection. J Infect Dis (2009) 200:337–46. doi: 10.1086/600120 19569965

[2] Olotu A Fegan G Wambua J Nyangweso G Leach A Lievens M. Seven-year efficacy of RTS,S/AS01 malaria vaccine among young African children. N Engl J Med (2016) 374:2519–29. doi: 10.1056/NEJMoa1515257 PMC4962898 27355532

[3] Datoo MS Natama MH Somé A Traoré O Rouamba T Bellamy D. Efficacy of a low-dose candidate malaria vaccine, R21 in adjuvant matrix-m, with seasonal administration to children in Burkina Faso: a randomised controlled trial. Lancet (2021) 397:1809–18. doi: 10.1016/S0140-6736(21)00943-0 PMC8121760 33964223

[4] Nussenzweig RS Vanderberg J Most H Orton C. Protective immunity produced by the injection of x-irradiated sporozoites of plasmodium berghei. Nature (1967) 216:160–2. doi: 10.1038/216160a0 6057225

[5] Clyde DF Most H McCarthy VC Vanderberg JP. Immunization of man against sporozite-induced falciparum malaria. Am J Med Sci (1973) 266:169–77. doi: 10.1097/00000441-197309000-00002 4583408

[6] Sissoko MS Healy SA Katile A Zaidi I Hu Z Kamate B. Safety and efficacy of a three-dose regimen of plasmodium falciparum sporozoite vaccine in adults during an intense malaria transmission season in Mali: a randomised, controlled phase 1 trial. Lancet Infect Dis (2022) 22:377–89. doi: 10.1016/S1473-3099(21)00332-7 PMC8981424 34801112

[7] Sissoko MS Healy SA Katile A Omaswa F Zaidi I Gabriel EE. Safety and efficacy of PfSPZ vaccine against plasmodium falciparum *via* direct venous inoculation in healthy malaria-exposed adults in Mali: a randomised, double-blind phase 1 trial. Lancet Infect Dis (2017) 17:498–509. doi: 10.1016/S1473-3099(17)30104-4 28216244 PMC6803168

[8] Tran TM Bijker EM Haks MC Ottenhoff THM Visser L Schats R. Whole-blood transcriptomic signatures induced during immunization by chloroquine prophylaxis and plasmodium falciparum sporozoites. Sci Rep (2019) 9:8386. doi: 10.1038/s41598-019-44924-7 31182757 PMC6557840

[9] Roestenberg M McCall M Hopman J Wiersma J Luty AJF van Gemert GJ. Protection against a malaria challenge by sporozoite inoculation. N Engl J Med (2009) 361:468–77. doi: 10.1056/NEJMoa0805832 19641203

[10] Vaughan AM Kappe SHI. Genetically attenuated malaria parasites as vaccines. Expert Rev Vaccines (2017) 16:765–7. doi: 10.1080/14760584.2017.1341835 28612631

[11] Goswami D Betz W Locham NK Parthiban C Brager C Schäfer C. A replication-competent late liver stage-attenuated human malaria parasite. JCI Insight (2020) 5. doi: 10.1172/jci.insight.135589 PMC7406309 32484795

[12] Mwakingwe-Omari A Healy SA Lane J Cook DM Kalhori S Wyatt C. Two chemoattenuated PfSPZ malaria vaccines induce sterile hepatic immunity. Nature (2021) 595:289–294. doi: 10.1038/s41586-021-03684-z PMC11127244 34194041

[13] Du Y Hertoghs N Duffy FJ Carnes J McDermott SM Neal ML. Systems analysis of immune responses to attenuated p. falciparum malaria sporozoite vaccination reveals excessive inflammatory signatures correlating with impaired immunity. PloS Pathog (2022) 18:e1010282. doi: 10.1371/journal.ppat.1010282 35108339 PMC8843222

[14] Sedegah M Hollingdale MR Ganeshan H Belmonte M Huang J Belmonte A. IMRAS-immunization with radiation-attenuated plasmodium falciparum sporozoites by mosquito bite: Cellular immunity to sporozoites, CSP, AMA1, TRAP and CelTOS. PloS One (2021) 16:e0256396. doi: 10.1371/journal.pone.0256396 34415964 PMC8378721

[15] Hickey B Teneza-Mora N Lumsden J Reyes S Sedegah M Garver L. IMRAS–a clinical trial of mosquito-bite immunization with live, radiation-attenuated p. falciparum sporozoites: Impact of immunization parameters on protective efficacy and generation of a repository of immunologic reagents. PloS One (2020) 15:1–30. doi: 10.1371/journal.pone.0233840 PMC7299375 32555601

[16] Gonzales SJ Reyes RA Braddom AE Batugedara G Bol S Bunnik EM. Naturally acquired humoral immunity against plasmodium falciparum malaria. Front Immunol (2020) 11:594653. doi: 10.3389/fimmu.2020.594653 33193447 PMC7658415

[17] McCallum FJ Persson KEM Fowkes FJI Reiling L Mugyenyi CK Richards JS. Differing rates of antibody acquisition to merozoite antigens in malaria: implications for immunity and surveillance. J Leukoc Biol (2017) 101:913–25. doi: 10.1189/jlb.5MA0716-294R PMC5346181 27837017

[18] Doolan DL Dobaño C Baird JK. Acquired immunity to malaria. Clin Microbiol Rev (2009) 22:13–36. doi: 10.1128/CMR.00025-08 19136431 PMC2620631

[19] Lautenbach MJ Yman V Silva CS Kadri N Broumou I Chan S. Systems analysis shows a role of cytophilic antibodies in shaping innate tolerance to malaria. Cell Rep (2022) 39:110709. doi: 10.1016/j.celrep.2022.110709 35443186

[20] Keitany GJ Kim KS Krishnamurty AT Hondowicz BD Hahn WO Dambrauskas N. Blood stage malaria disrupts humoral immunity to the pre-erythrocytic stage circumsporozoite protein. Cell Rep (2016) 17:3193–205. doi: 10.1016/j.celrep.2016.11.060 PMC5476299 28009289

[21] Wilson NS Behrens GMN Lundie RJ Smith CM Waithman J Young L. Systemic activation of dendritic cells by toll-like receptor ligands or malaria infection impairs cross-presentation and antiviral immunity. Nat Immunol (2006) 7:165–72. doi: 10.1038/ni1300 16415871

[22] Dobin A Gingeras TR. Mapping RNA-seq reads with STAR. current protocols in bioinformatics. Hoboken, NJ, USA: John Wiley & Sons, Inc (2015). doi: 10.1002/0471250953.bi1114s51 PMC4631051 26334920

[23] Putri GH Anders S Pyl PT Pimanda JE Zanini F. Analysing high-throughput sequencing data in Python with HTSeq 2.0. Bioinformatics (2022) 38(10):2943–2945. doi: 10.1093/bioinformatics/btac166 PMC9113351 35561197

[24] Zhang Y Parmigiani G Johnson WE. ComBat-seq: batch effect adjustment for RNA-seq count data. NAR Genomics Bioinforma (2020) 2:lqaa078. doi: 10.1093/nargab/lqaa078 PMC7518324 33015620

[25] Ritchie ME Phipson B Wu D Hu Y Law CW Shi W. Limma powers differential expression analyses for RNA-sequencing and microarray studies. Nucleic Acids Res (2015) 43:e47. doi: 10.1093/nar/gkv007 25605792 PMC4402510

[26] Law CW Chen Y Shi W Smyth GK. Voom: precision weights unlock linear model analysis tools for RNA-seq read counts. Genome Biol (2014) 15:R29. doi: 10.1186/gb-2014-15-2-r29 24485249 PMC4053721

[27] Hoffman GE Roussos P. Dream: powerful differential expression analysis for repeated measures designs. Bioinformatics (2021) 37:192–201. doi: 10.1093/bioinformatics/btaa687 32730587 PMC8055218

[28] Korotkevich G Sukhov V Budin N Shpak B Artyomov MN Sergushichev A. Fast gene set enrichment analysis. bioRxiv (2021), 60012. doi: 10.1101/060012

[29] Rinchai D Roelands J Toufiq M Hendrickx W Altman MC Bedognetti D. BloodGen3Module: blood transcriptional module repertoire analysis and visualization using r. Bioinformatics (2021) 37:2382–9. doi: 10.1093/bioinformatics/btab121 PMC8388021 33624743

[30] Mair F Prlic M. OMIP-044: 28-color immunophenotyping of the human dendritic cell compartment. Cytometry A (2018) 93:402–5. doi: 10.1002/cyto.a.23331 PMC6785241 29356334

[31] Hertoghs N Schwedhelm KV Stuart KD McElrath MJ De Rosa SC. OMIP-064: A 27-color flow cytometry panel to detect and characterize human NK cells and other innate lymphoid cell subsets And γδ T cells. Cytometry A (2020) 97:1019–23. doi: 10.1002/cyto.a.24031 PMC8485754 32415811

[32] Bates D Mächler M Bolker B Walker S. Fitting linear mixed-effects models using lme4. J Stat Software (2015) 67:1–48. doi: 10.18637/jss.v067.i01

[33] Coley AM Gupta A Murphy VJ Bai T Kim H Foley M. Structure of the malaria antigen AMA1 in complex with a growth-inhibitory antibody. PloS Pathog (2007) 3:1308–19. doi: 10.1371/journal.ppat.0030138 PMC2323298 17907804

[34] Akhouri RR Bhattacharyya A Pattnaik P Malhotra P Sharma A. Structural and functional dissection of the adhesive domains of plasmodium falciparum thrombospondin-related anonymous protein (TRAP). Biochem J (2004) 379:815–22. doi: 10.1042/BJ20031500 PMC1224115 14741048

[35] Coppi A Natarajan R Pradel G Bennett BL James ER Roggero MA. The malaria circumsporozoite protein has two functional domains, each with distinct roles as sporozoites journey from mosquito to mammalian host. J Exp Med (2011) 208:341–56. doi: 10.1084/jem.20101488 PMC3039851 21262960

[36] Seder RA Chang LJ Enama ME Zephir KL Sarwar UN Gordon IJ. Protection against malaria by intravenous immunization with a nonreplicating sporozoite vaccine. Science (2013) 341:1359–65. doi: 10.1126/science.1241800 23929949

[37] Lyke KE Ishizuka AS Berry AA Chakravarty S DeZure A Enama ME. Attenuated PfSPZ vaccine induces strain-transcending T cells and durable protection against heterologous controlled human malaria infection. Proc Natl Acad Sci U S A (2017) 114:2711–6. doi: 10.1073/pnas.1615324114 PMC5347610 28223498

[38] Duffy FJ Du Y Carnes J Epstein JE Hoffman SL Abdulla S. Early whole blood transcriptional responses to radiation-attenuated plasmodium falciparum sporozoite vaccination in malaria naïve and malaria pre-exposed adult volunteers. Malar J (2021) 20:308. doi: 10.1186/s12936-021-03839-3 34243763 PMC8267772

[39] Jongo SA Shekalaghe SA Church LWP Ruben AJ Schindler T Zenklusen I. Safety, immunogenicity, and protective efficacy against controlled human malaria infection of plasmodium falciparum sporozoite vaccine in Tanzanian adults. Am J Trop Med Hyg (2018) 99:338–49. doi: 10.4269/ajtmh.17-1014 PMC6090339 29943719

[40] Suscovich TJ Fallon JK Das J Demas AR Crain J Linde CH. Mapping functional humoral correlates of protection against malaria challenge following RTS,S/AS01 vaccination. Sci Transl Med (2020) 12. doi: 10.1126/SCITRANSLMED.ABB4757 32718991

[41] Cockburn IA Seder RA. Malaria prevention: from immunological concepts to effective vaccines and protective antibodies. Nat Immunol (2018) 19:1199–211. doi: 10.1038/s41590-018-0228-6 30333613

[42] Gaudinski MR Berkowitz NM Idris AH Coates EE Holman LA Mendoza F. A monoclonal antibody for malaria prevention. N Engl J Med (2021), 385(9):803–814. doi: 10.1056/NEJMoa2034031 34379916 PMC8579034

[43] Epstein JE Tewari K Lyke KE Sim BKL Billingsley PF Laurens MB. Live attenuated malaria vaccine designed to protect through hepatic CD8^+^ T cell immunity. Science (2011) 334:475–80. doi: 10.1126/science.1211548 21903775

[44] Minkah NK Wilder BK Sheikh AA Martinson T Wegmair L Vaughan AM. Innate immunity limits protective adaptive immune responses against pre-erythrocytic malaria parasites. Nat Commun (2019) 10:1–14. doi: 10.1038/s41467-019-11819-0 31477704 PMC6718385

[45] Jeon J-H Hong C-W Kim EY Lee JM. Current understanding on the metabolism of neutrophils. Immune Netw (2020) 20:e46. doi: 10.4110/in.2020.20.e46 33425431 PMC7779868

[46] Kelly B O’Neill LAJ. Metabolic reprogramming in macrophages and dendritic cells in innate immunity. Cell Res (2015) 25:771–84. doi: 10.1038/cr.2015.68 PMC4493277 26045163

[47] Viola A Munari F Sánchez-Rodríguez R Scolaro T Castegna A. The metabolic signature of macrophage responses. Front Immunol (2019) 10:1462. doi: 10.3389/fimmu.2019.01462 31333642 PMC6618143

[48] Jones RG Thompson CB. Revving the engine: Signal transduction fuels T cell activation. Immunity (2007) 27:173–8. doi: 10.1016/j.immuni.2007.07.008 17723208

[49] Calzetti F Tamassia N Micheletti A Finotti G Bianchetto-Aguilera F Cassatella MA. Human dendritic cell subset 4 (DC4) correlates to a subset of CD14dim/-CD16++ monocytes. J Allergy Clin Immunol (2018) 141:2276–2279.e3. doi: 10.1016/j.jaci.2017.12.988 29366702

[50] Dutertre C-A Becht E Irac SE Khalilnezhad A Narang V Khalilnezhad S. Single-cell analysis of human mononuclear phagocytes reveals subset-defining markers and identifies circulating inflammatory dendritic cells. Immunity (2019) 51:573–589.e8. doi: 10.1016/j.immuni.2019.08.008 31474513

[51] Mpina M Maurice NJ Yajima M Slichter CK Miller HW Dutta M. Controlled human malaria infection leads to long-lasting changes in innate and innate-like lymphocyte populations. J Immunol (2017) 199:107–18. doi: 10.4049/jimmunol.1601989 PMC5528886 28576979

[52] Behr C Dubois P. Preferential expansion of V gamma 9 V delta 2 T cells following stimulation of peripheral blood lymphocytes with extracts of plasmodium falciparum. Int Immunol (1992) 4:361–6. doi: 10.1093/intimm/4.3.361 1533150

[53] Dantzler KW Jagannathan P. γδ T cells in antimalarial immunity: New insights into their diverse functions in protection and tolerance. Front Immunol (2018) 9:2445. doi: 10.3389/fimmu.2018.02445 30405634 PMC6206268

[54] Zaidi I Diallo H Conteh S Robbins Y Kolasny J Orr-Gonzalez S. γδ T cells are required for the induction of sterile immunity during irradiated sporozoite vaccinations. J Immunol (2017) 199:3781–8. doi: 10.4049/jimmunol.1700314 PMC5698172 29079696

[55] Pamplona A Silva-Santos B. γδ T cells in malaria: a double-edged sword. FEBS J (2021) 288:1118–29. doi: 10.1111/febs.15494 PMC7983992 32710527

[56] Jagannathan P Kim CC Greenhouse B Nankya F Bowen K Eccles-James I. Loss and dysfunction of Vδ2^+^ γδ T cells are associated with clinical tolerance to malaria. Sci Transl Med (2014) 6:251ra117. doi: 10.1126/scitranslmed.3009793 PMC4198150 25163477

[57] Kurup SP Anthony SM Hancox LS Vijay R Pewe LL Moioffer SJ. Monocyte-derived CD11c+ cells acquire plasmodium from hepatocytes to prime CD8 T cell immunity to liver-stage malaria. Cell Host Microbe (2019) 25:565–577.e6. doi: 10.1016/j.chom.2019.02.014 30905437 PMC6459714

[58] Veeranki S Duan X Panchanathan R Liu H Choubey D. IFI16 protein mediates the anti-inflammatory actions of the type-I interferons through suppression of activation of caspase-1 by inflammasomes. PloS One (2011) 6:e27040. doi: 10.1371/journal.pone.0027040 22046441 PMC3203938

[59] Kazmin D Nakaya HI Lee EK Johnson MJ van der Most R van den Berg RA. Systems analysis of protective immune responses to RTS,S malaria vaccination in humans. Proc Natl Acad Sci (2017) 114:2425–30. doi: 10.1073/pnas.1621489114 PMC5338562 28193898

[60] Royo J Rahabi M Kamaliddin C Ezinmegnon S Olagnier D Authier H. Changes in monocyte subsets are associated with clinical outcomes in severe malarial anaemia and cerebral malaria. Sci Rep (2019) 9:1–13. doi: 10.1038/s41598-019-52579-7 31772386 PMC6879635

[61] Collin M Bigley V. Human dendritic cell subsets: an update. Immunology (2018) 154:3–20. doi: 10.1111/imm.12888 29313948 PMC5904714

[62] Ma DY Clark EA. The role of CD40 and CD154/CD40L in dendritic cells. Semin Immunol (2009) 21:265–72. doi: 10.1016/j.smim.2009.05.010 PMC2749083 19524453

[63] Murray SA Mohar I Miller JL Brempelis KJ Vaughan AM Kappe SHI. CD40 is required for protective immunity against liver stage plasmodium infection. J Immunol (2015) 194:2268–79. doi: 10.4049/jimmunol.1401724 PMC4340756 25646303

[64] Götz A Tang MS Ty MC Arama C Ongoiba A Doumtabe D. Atypical activation of dendritic cells by plasmodium falciparum. Proc Natl Acad Sci U S A (2017) 114:E10568–77. doi: 10.1073/pnas.1708383114 PMC5724257 29162686

[65] Neal ML Duffy FJ Du Y Aitchison JD Stuart KD. Preimmunization correlates of protection shared across malaria vaccine trials in adults. J Vaccines (2022) 7. doi: 10.1038/s41541-021-00425-1 PMC8760258 35031601

[66] Fourati S Tomalin L Mule M Chawla D Gerritson B Rychkov D. An innate immune activation state prior to vaccination predicts responsiveness to multiple vaccines Authors : Affiliations : Abstract : Introduction. Biorxiv (2021). doi: 10.1101/2021.09.26.461847

[67] Weiss G Ganz T Goodnough LT. Anemia of inflammation. Blood (2019) 133:40–50. doi: 10.1182/blood-2018-06-856500 30401705 PMC6536698

[68] Essers MAG Offner S Blanco-Bose WE Waibler Z Kalinke U Duchosal MA. IFNalpha activates dormant haematopoietic stem cells in vivo. Nature (2009) 458:904–8. doi: 10.1038/nature07815 19212321

[69] Demerdash Y Kain B Essers MAG King KY. Yin and yang: The dual effects of interferons on hematopoiesis. Exp Hematol (2021) 96:1–12. doi: 10.1016/j.exphem.2021.02.002 33571568 PMC8919039

[70] Andrade BB Araújo-Santos T Luz NF Khouri R Bozza MT Camargo LMA. Heme impairs prostaglandin E2 and TGF-beta production by human mononuclear cells *via* Cu/Zn superoxide dismutase: insight into the pathogenesis of severe malaria. J Immunol (2010) 185:1196–204. doi: 10.4049/jimmunol.0904179 20562262

[71] Perkins DJ Kremsner PG Weinberg JB. Inverse relationship of plasma prostaglandin E2 and blood mononuclear cell cyclooxygenase-2 with disease severity in children with plasmodium falciparum malaria. J Infect Dis (2001) 183:113–8. doi: 10.1086/317660 11076710

