# Supplementary figures and images for: Longitudinal immune profiling after radiation-attenuated sporozoite vaccination reveals coordinated immune processes correlated with malaria protection
Source: Front Immunol. 2022 Dec 15;13:1042741. doi: 10.3389/fimmu.2022.1042741 (PMC9798120; doi:10.3389/fimmu.2022.1042741)

**S002 P**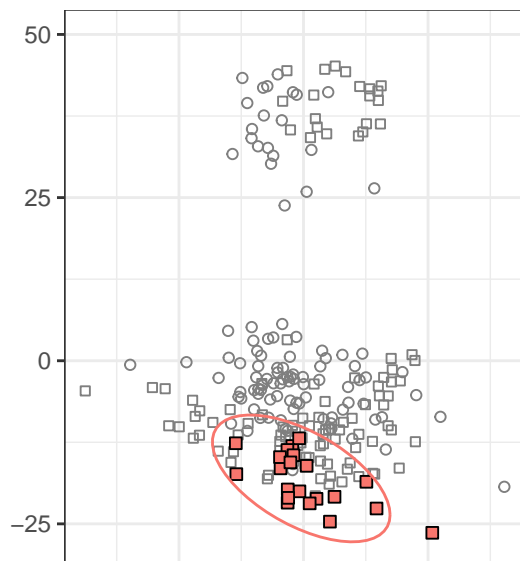**S005 NP**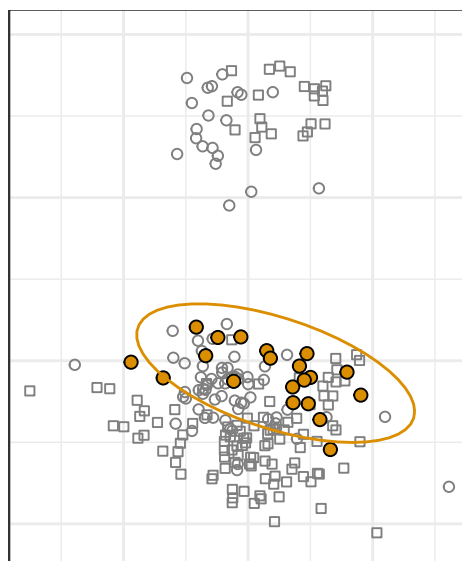**S021 NP**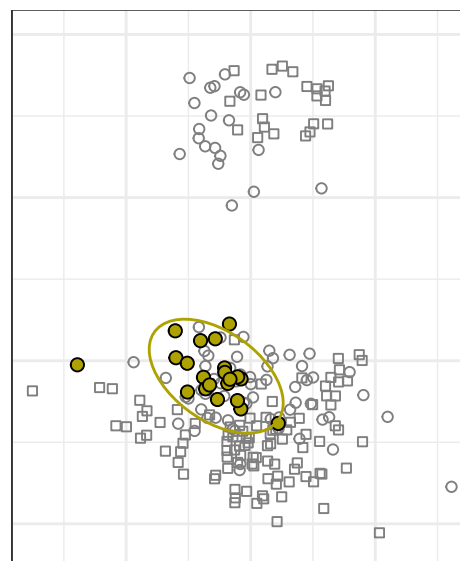**S033 NP**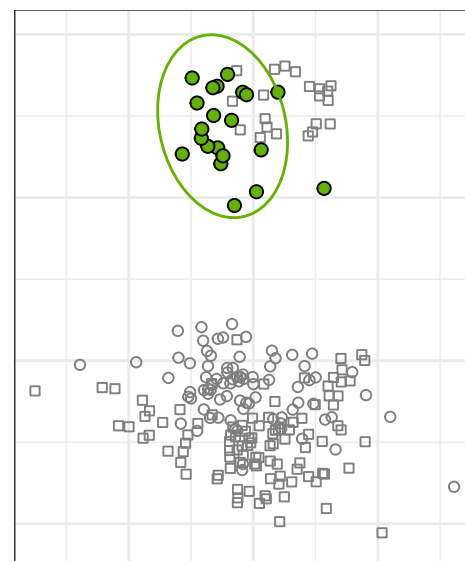**S039 P**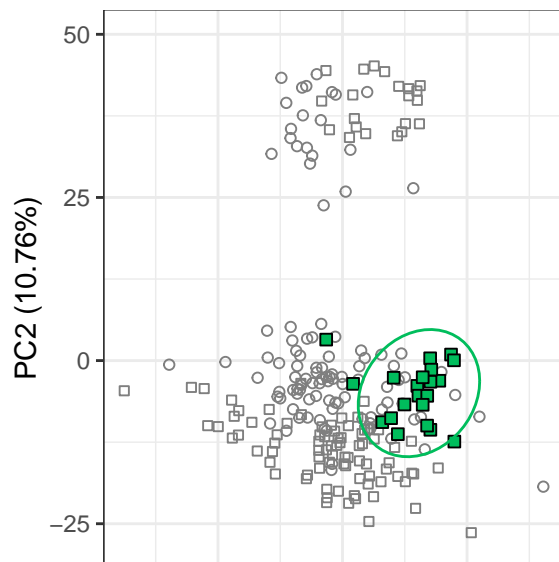**S045 P**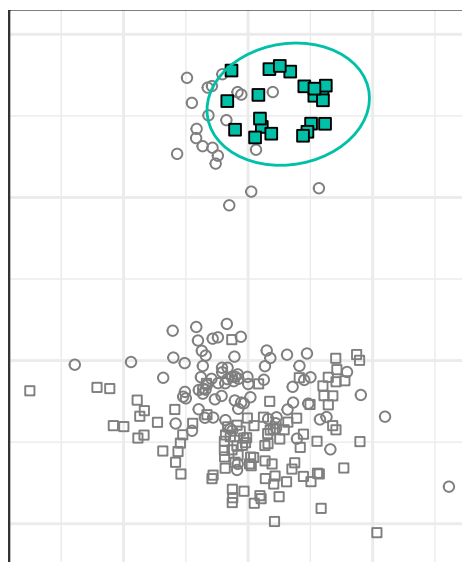**S057 P**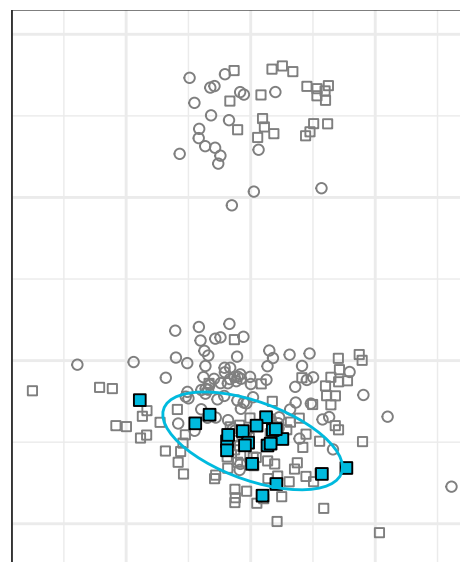**S060 P**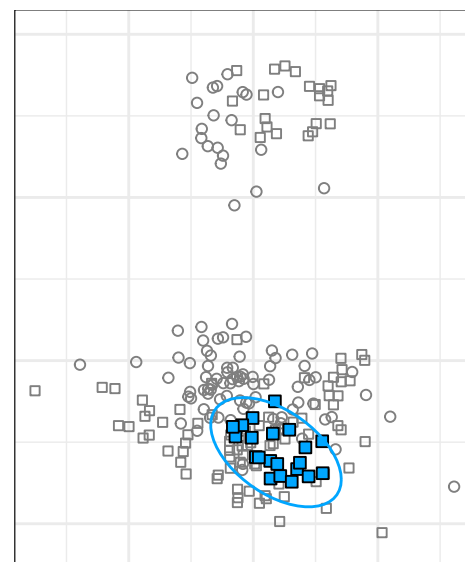**S061 NP**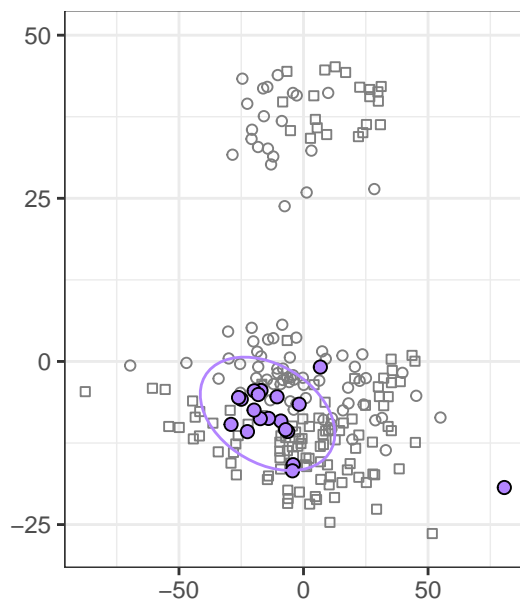**S071 NP**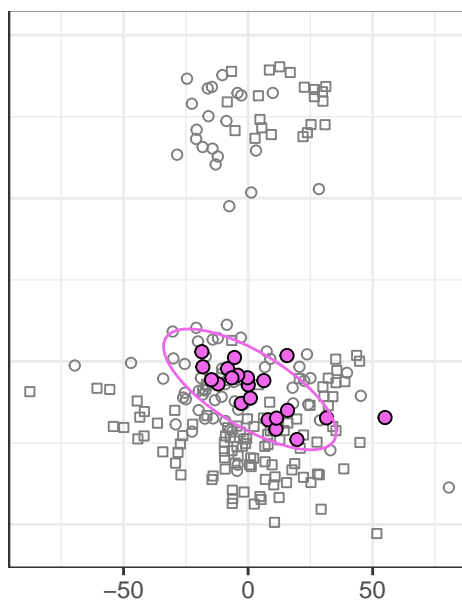**S078 P**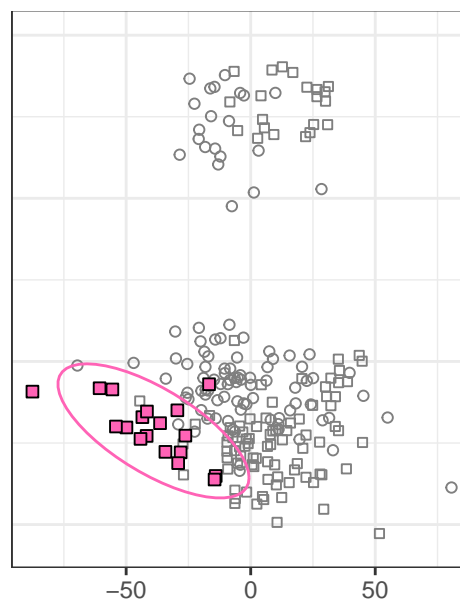

PC1 (16.62%)

Supplement: Supplementary Figure 1 — Principal component analysis plots of normalized whole blood gene expression data. Each panel shows colored points corresponding to all timepoints from a single participant with samples from other study participants shown in grey. [file Image_1.pdf]

**A**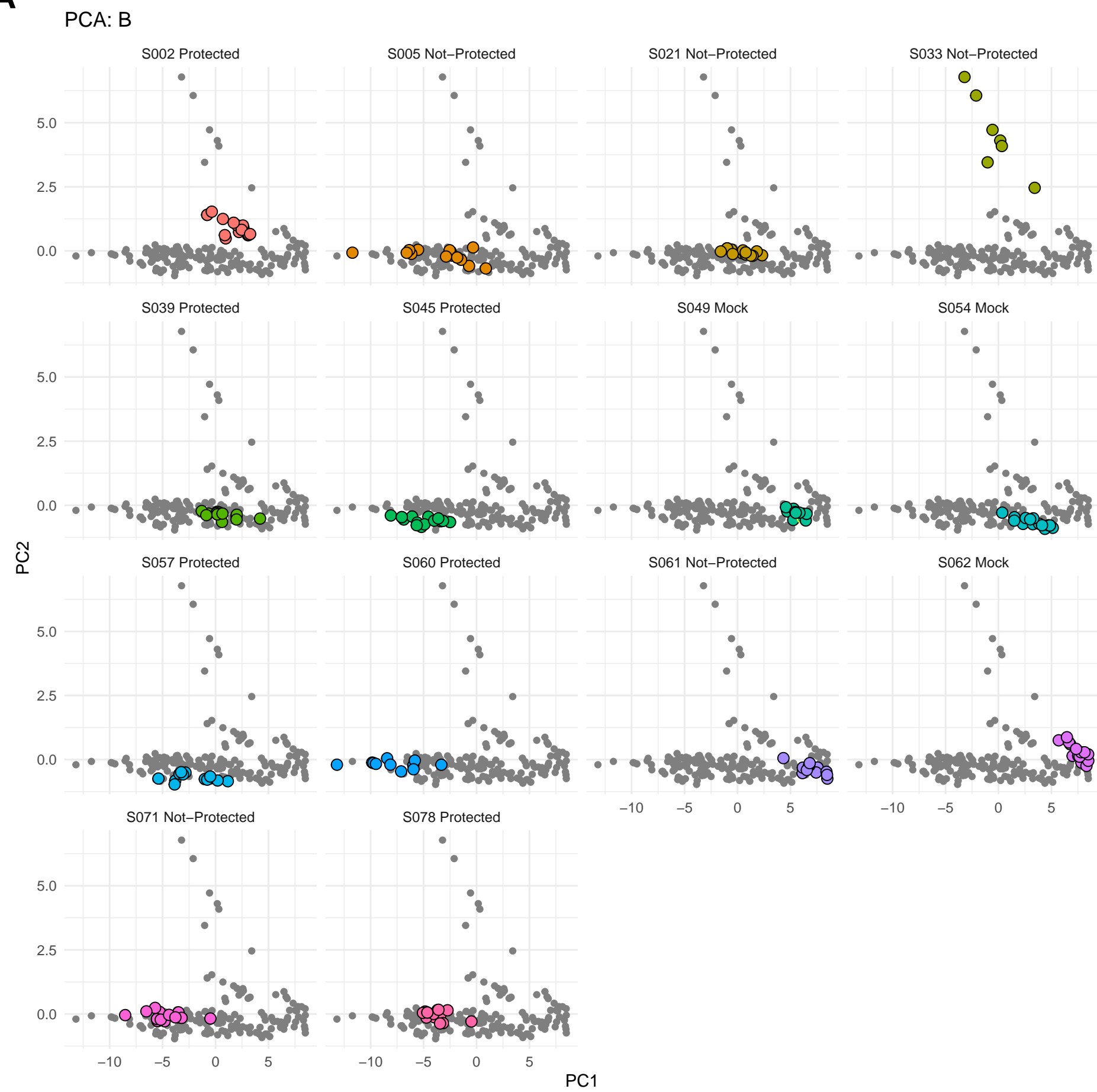**B**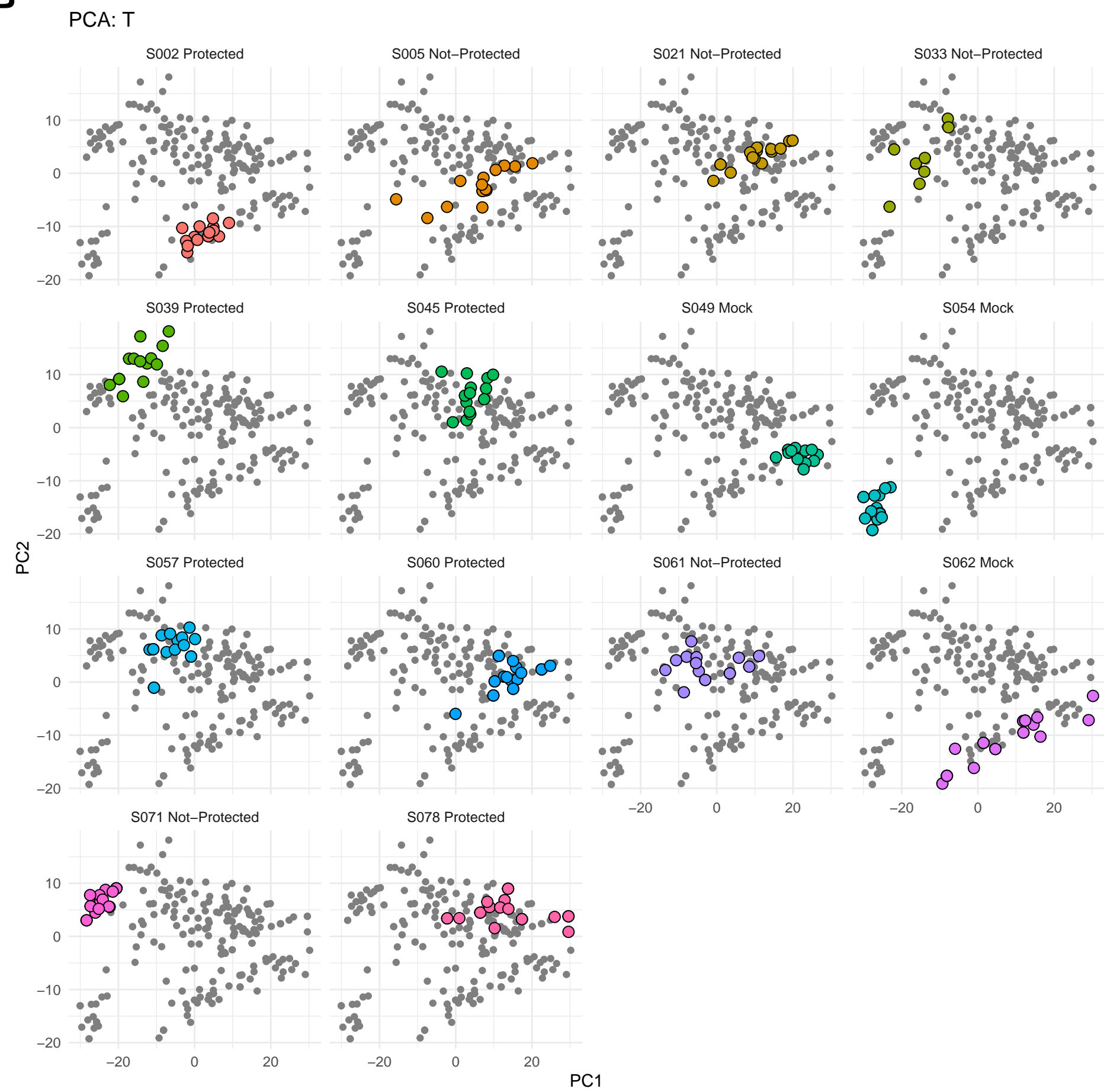**C**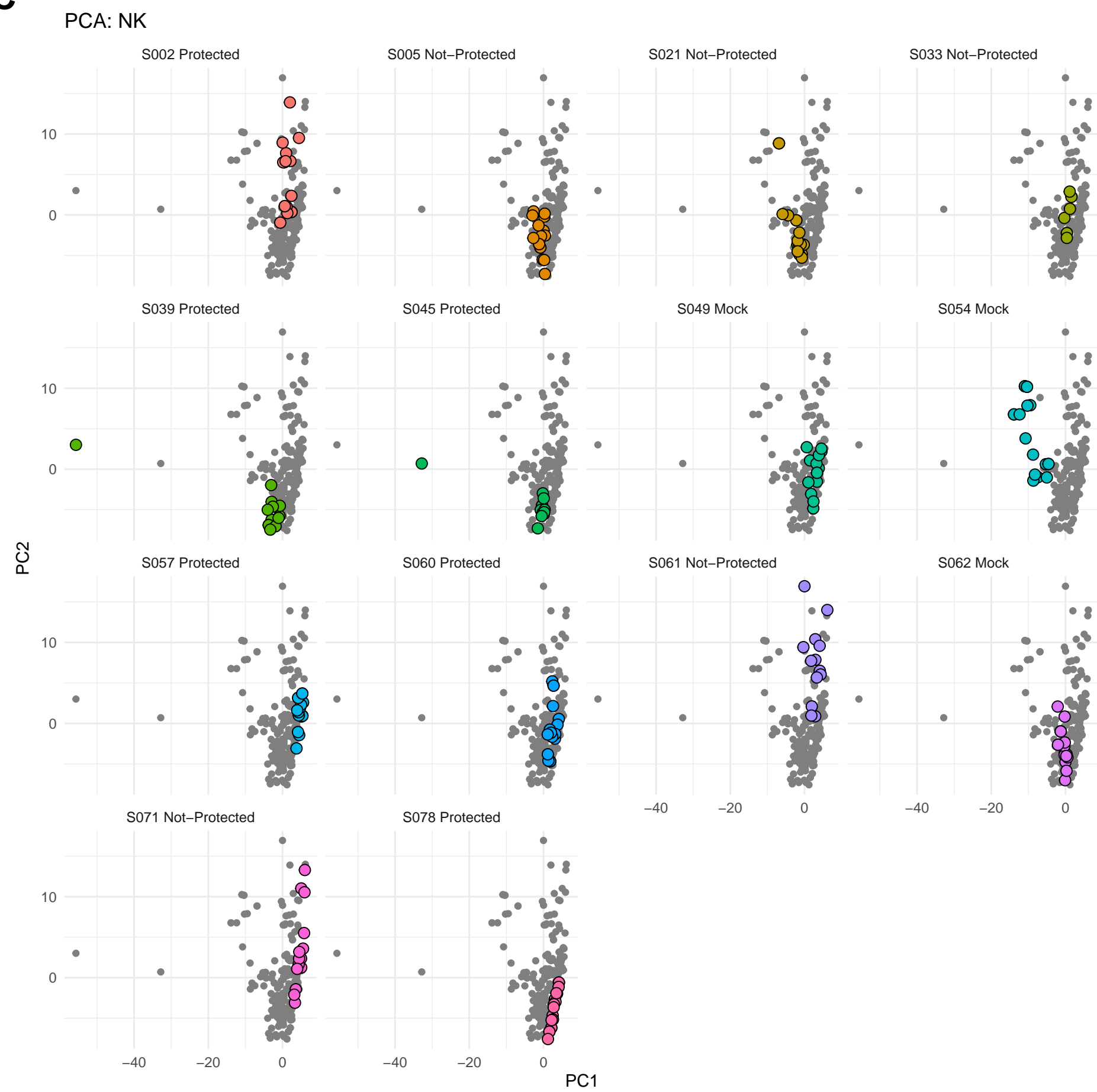**D**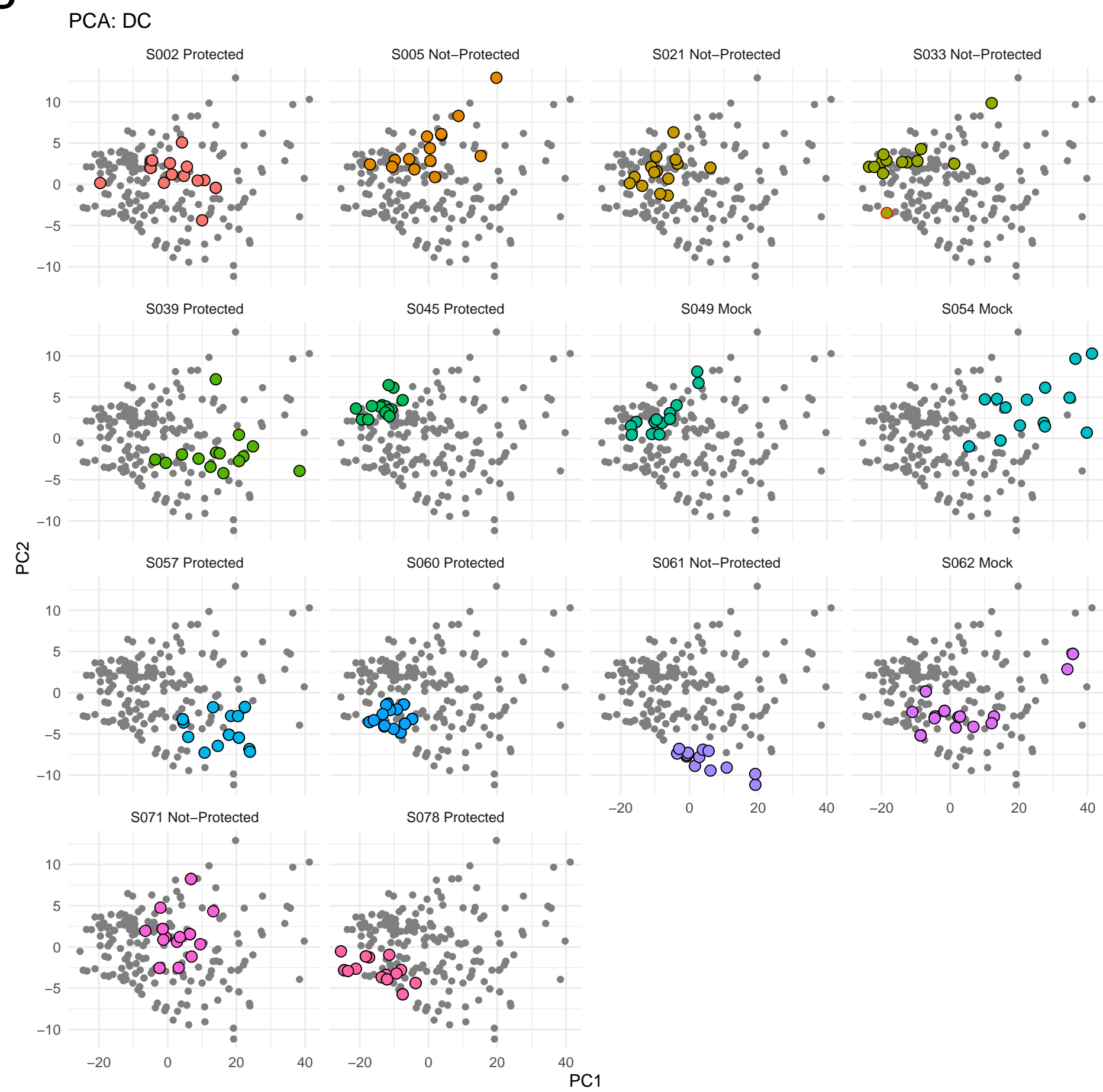

Supplement: Supplementary Figure 2 — Principal component analysis plots of cell-type profiles determined by flow cytometry. Each plot panel shows the first two principal components per-sample from flow panels for B-cells; CD3+ T cells; NK and invariant T-cells; and DCs and other antigen presenting cells respectively. Each sub-panel is labelled and colored to highlight a single study participant, with the remaining samples shown in grey. [file Image_2.pdf]
