# Supplementary material for: Longitudinal immune profiling after radiation-attenuated sporozoite vaccination reveals coordinated immune processes correlated with malaria protection
Source: Front Immunol. 2022 Dec 15;13:1042741. doi: 10.3389/fimmu.2022.1042741 (PMC9798120; doi:10.3389/fimmu.2022.1042741)

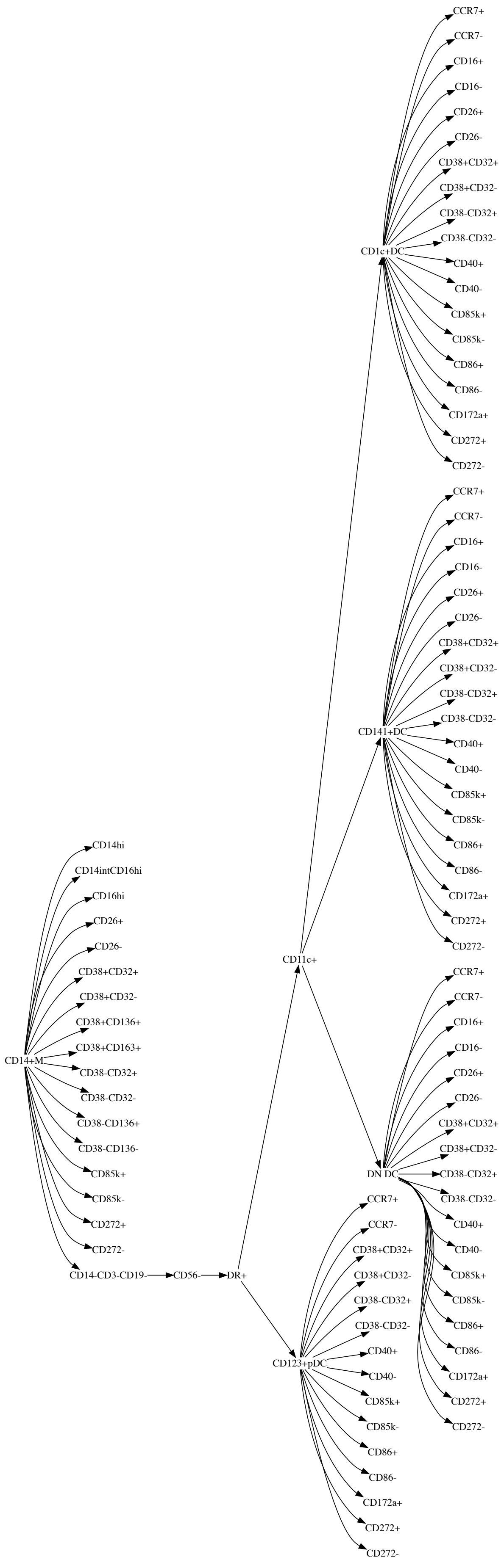

DC/APC gating

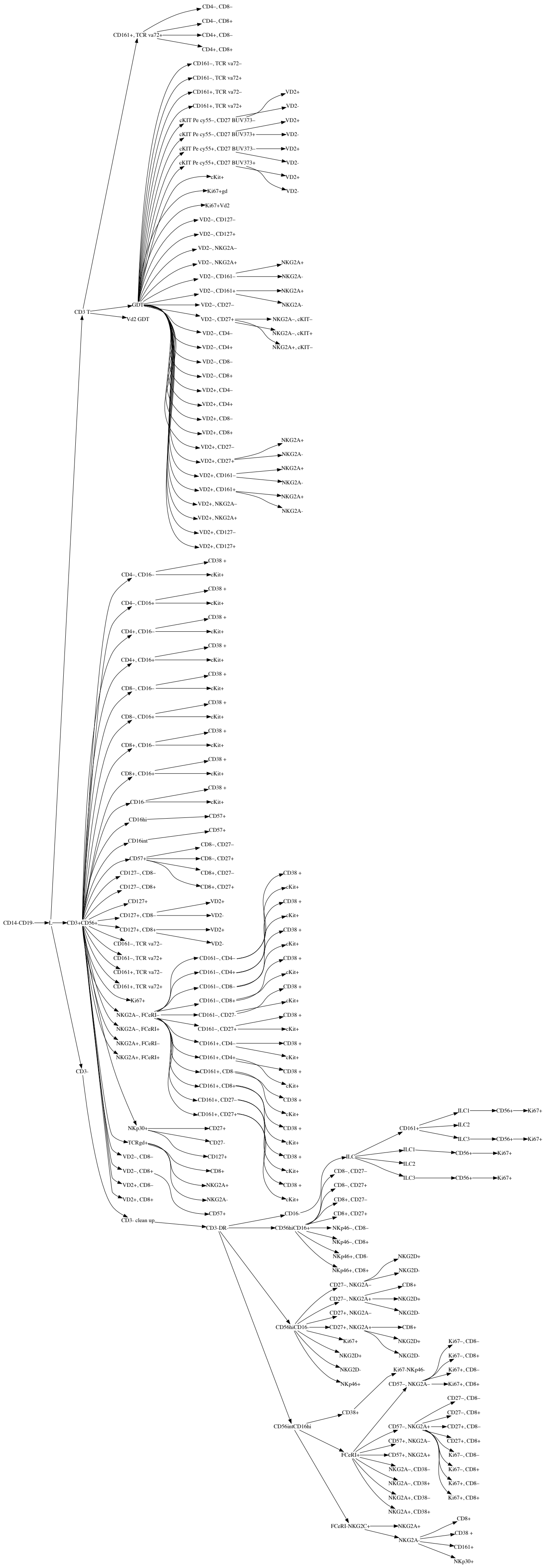

NK/ILC gating

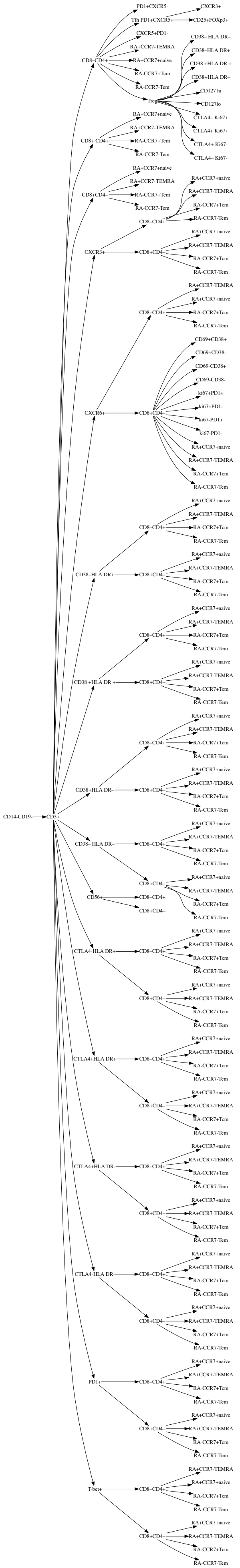

T cell gating

T cell gating

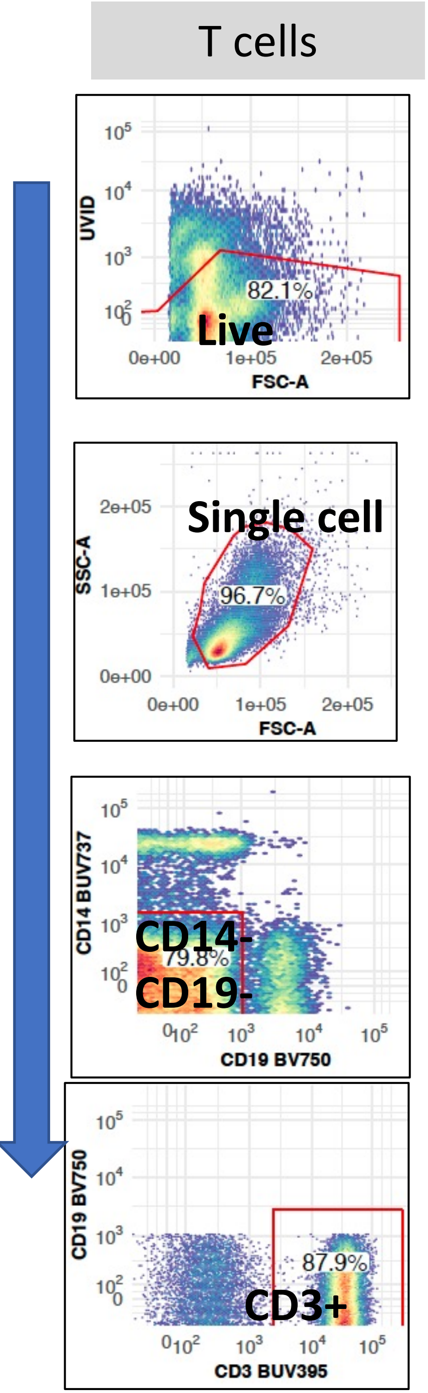

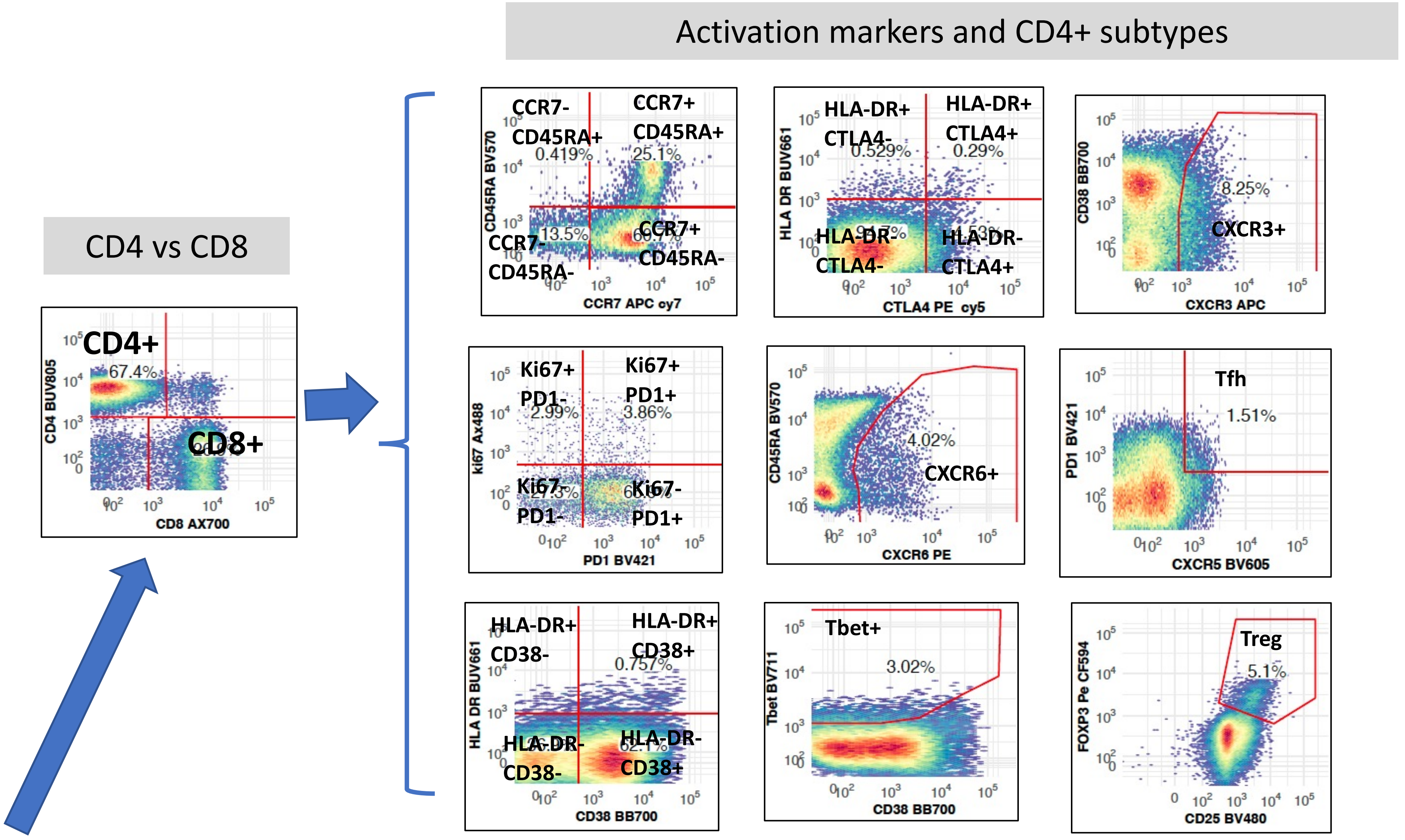

B cell gating

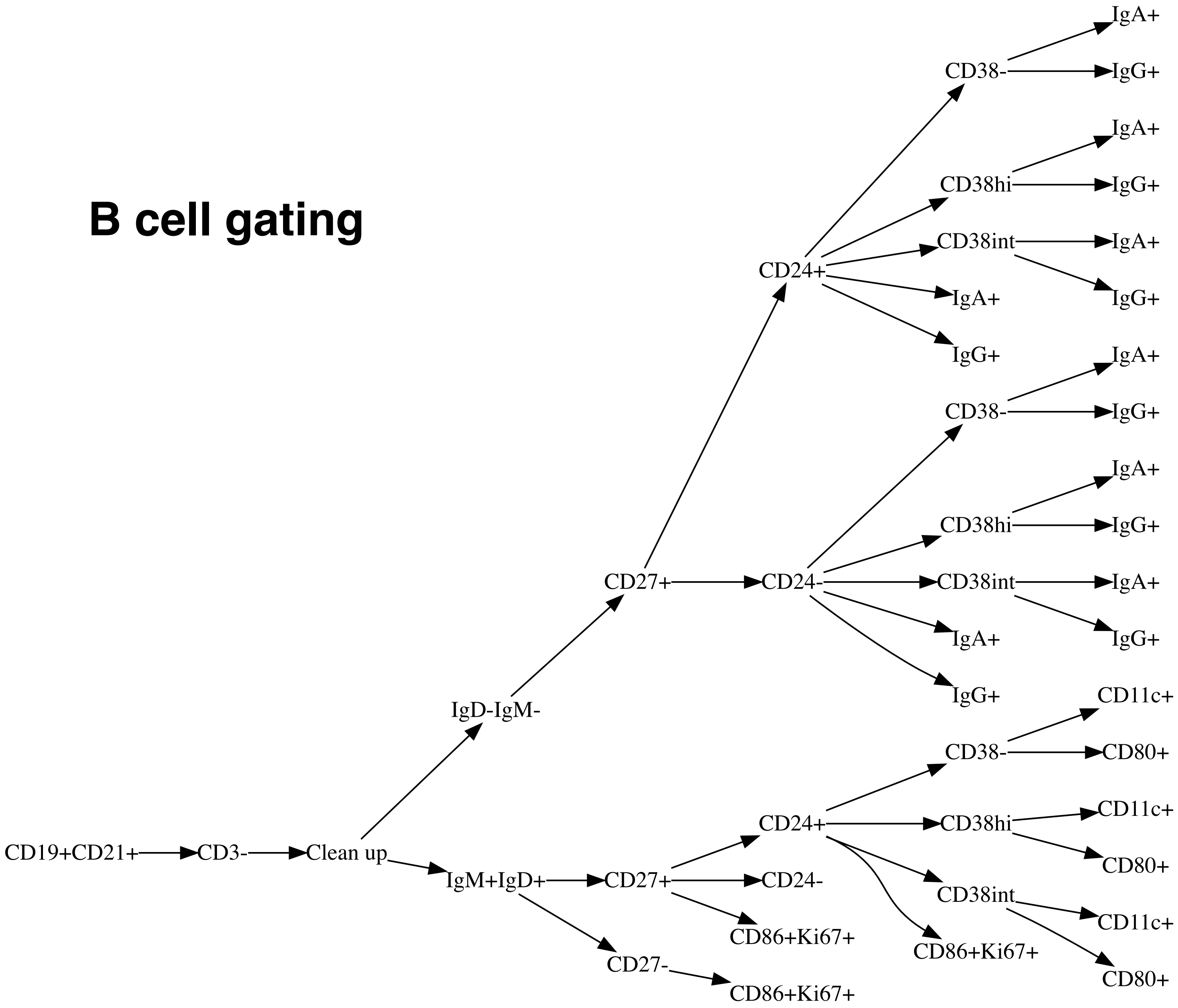

B cell gating

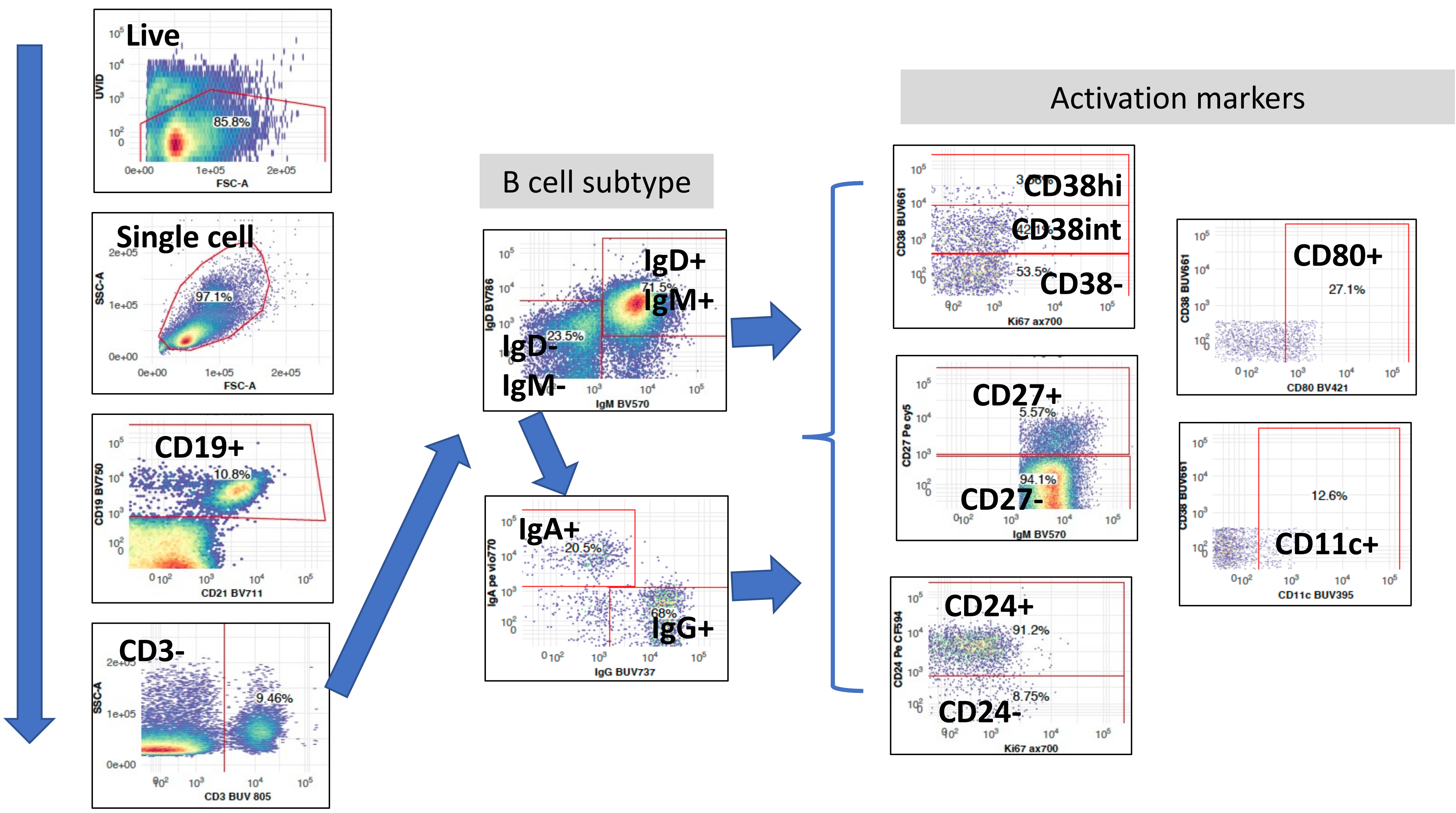

Supplement: Supplementary Data Sheet 1 — Limma-dream results table of significant (FDR < 0.05) vaccine-induced genes. [file DataSheet_1.zip › Datafile-S-2.pdf]
